# Supplementary material for: Spatio-temporal variability of eDNA signal and its implication for fish monitoring in lakes
Source: PLoS One. 2022 Aug 12;17(8):e0272660. doi: 10.1371/journal.pone.0272660 (PMC9374266; doi:10.1371/journal.pone.0272660)
Supplement: S1 Table — (DOCX) [file pone.0272660.s001.docx]

**S1. Table. CEN gillnets campaigns for the lakes Aiguebelette, Serre-Ponçon and Etang des Aulnes.** The table detailed the number of the individuals caugth per species and per campaign (date) and the number of gillnets (used) in which they were recorded.

| **Location** | **Aiguebelette** | | | | | | | | | | | |
| --- | --- | --- | --- | --- | --- | --- | --- | --- | --- | --- | --- | --- |
| **Campaign** | 10/2009 | | | | 09/2014 | | | | 10/2020 | | | |
| **Type of gillnet** | Benthic | | Pelagic | | Benthic | | Pelagic | | Benthic | | Pelagic | |
| **Species** | Individuals | Nets (58) | Individuals | Nets (22) | Individuals | Nets (58) | Individuals | Nets (22) | Individuals | Nets (56) | Individuals | Nets (18) |
| *Abramis brama* | 7 | 3 |  |  | 1 | 1 | 1 | 1 | 7 | 4 |  |  |
| *Alburnus alburnus* |  |  |  |  |  |  |  |  |  |  |  |  |
| *Blicca bjoerkna* |  |  |  |  |  |  |  |  |  |  |  |  |
| *Carassius carassius* |  |  |  |  |  |  |  |  |  |  |  |  |
| *Coregonus sp* | 15 | 5 | 117 | 8 | 8 | 6 | 45 | 9 | 15 | 6 | 67 | 9 |
| *Cyprinus carpio* |  |  |  |  | 1 | 1 |  |  |  |  |  |  |
| *Esox lucius* | 5 | 4 |  |  | 7 | 6 |  |  | 2 | 2 |  |  |
| *Gobio gobio* | 24 | 10 |  |  | 11 | 5 |  |  | 64 | 8 |  |  |
| *Lepomis gibbosus* | 4 | 3 |  |  | 1 | 1 |  |  | 12 | 6 |  |  |
| *Leuciscus leuciscus* |  |  |  |  |  |  |  |  | 11 | 4 |  |  |
| *Parachondrostoma toxostoma* |  |  |  |  |  |  |  |  |  |  |  |  |
| *Perca fluviatilis* | 268 | 26 |  |  | 1132 | 30 | 5 | 2 | 514 | 30 | 1 | 1 |
| *Rutilus rutilus* | 411 | 30 | 53 | 3 | 344 | 28 | 61 | 2 | 354 | 24 | 44 | 4 |
| *Salmo trutta* |  |  |  |  |  |  |  |  |  |  |  |  |
| *Salvelinus umbla* | 6 | 4 | 2 | 2 | 10 | 4 | 2 | 2 |  |  |  |  |
| *Sander lucioperca* | 15 | 7 |  |  | 2 | 2 |  |  | 3 | 3 |  |  |
| *Scardinius erythrophthalmus* | 33 | 8 |  |  | 34 | 8 |  |  | 186 | 11 | 2 | 2 |
| *Silurus glanis* |  |  |  |  |  |  |  |  |  |  |  |  |
| *Squalius cephalus* | 9 | 7 |  |  | 3 | 3 |  |  | 1 | 1 |  |  |
| *Tinca tinca* | 1 | 1 |  |  | 3 | 3 |  |  | 1 | 1 |  |  |
| **Species** | 12 |  | 3 |  | 13 |  | 5 |  | 12 |  | 4 |  |
| **Individuals** | 798 |  | 172 |  | 1557 |  | 114 |  | 1170 |  | 114 |  |

| **Location** | **Serre-Ponçon** | | | | | | | | **AUL13** | | | |
| --- | --- | --- | --- | --- | --- | --- | --- | --- | --- | --- | --- | --- |
| **Campaign** | 09/2011 | | | | 09/2017 | | | | 07/2011 | | 07/2015 | |
| **Type of gillnet** | Benthic | | Pelagic | | Benthic | | Pelagic | | Benthic | | Benthic | |
| **Species** | Individuals | Nets (68) | Individuals | Nets (42) | Individuals | Nets (64) | Individuals | Nets (24) | Individuals | Nets (16) | Individuals | Nets (16) |
| *Abramis brama* | 5 | 4 |  |  | 5 | 5 |  |  | 49 | 7 (16) |  |  |
| *Alburnus alburnus* | 107 | 16 | 34 | 9 | 154 | 22 | 18 | 3 |  |  |  |  |
| *Blicca bjoerkna* |  |  |  |  | 1 | 1 |  |  | 3 | 3 (16) | 97 | 12 (16) |
| *Carassius carassius* |  |  |  |  |  |  |  |  | 2 | 2 (16) |  |  |
| *Coregonus sp* | 17 | 10 | 17 | 10 |  |  | 17 | 4 |  |  |  |  |
| *Cyprinus carpio* |  |  |  |  |  |  |  |  |  |  | 1 | 1 (16) |
| *Esox lucius* | 4 | 4 |  |  | 1 | 1 |  |  | 3 | 2 (16) | 12 | 6 (16) |
| *Gobio gobio* | 305 | 51 | 1 | 1 | 251 | 44 |  |  |  |  |  |  |
| *Lepomis gibbosus* |  |  |  |  |  |  |  |  | 12 | 4 (16) | 225 | 9 (16) |
| *Leuciscus leuciscus* |  |  |  |  |  |  |  |  |  |  |  |  |
| *Parachondrostoma toxostoma* | 53 | 13 |  |  | 82 | 12 |  |  |  |  |  |  |
| *Perca fluviatilis* | 519 | 43 | 1 | 1 | 684 | 51 | 22 | 7 | 208 | 13 (16) | 1397 | 14 (16) |
| *Rutilus rutilus* | 243 | 41 | 7 | 5 | 186 | 47 | 8 | 6 | 339 | 10 (16) | 816 | 15 (16) |
| *Salmo trutta* | 6 | 6 | 6 | 6 | 2 | 2 | 2 | 1 |  |  |  |  |
| *Salvelinus umbla* | 1 | 1 |  |  |  |  |  |  |  |  |  |  |
| *Sander lucioperca* |  |  |  |  |  |  |  |  | 2134 | 16 (16) | 135 | 9 (16) |
| *Scardinius erythrophthalmus* | 2 | 2 |  |  |  |  |  |  | 150 | 8 (16) | 172 | 10 (16) |
| *Silurus glanis* |  |  |  |  |  |  |  |  | 1 | 1 (16) | 5 | 5 (16) |
| *Squalius cephalus* | 9 | 8 |  |  | 16 | 15 |  |  |  |  |  |  |
| *Tinca tinca* |  |  |  |  | 1 | 1 |  |  | 46 | 8 (16) | 553 | 12 (16) |
| **Species** | 12 |  | 6 |  | 11 |  | 5 |  | 11 |  | 10 |  |
| **Individuals** | 1271 |  | 66 |  | 1383 |  | 67 |  | 2947 |  | 3413 |  |
