# Supplementary material for: Spatio-temporal variability of eDNA signal and its implication for fish monitoring in lakes
Source: PLoS One. 2022 Aug 12;17(8):e0272660. doi: 10.1371/journal.pone.0272660 (PMC9374266; doi:10.1371/journal.pone.0272660)
Supplement: S1 Text — (DOCX) [file pone.0272660.s002.docx]

**S2. Text. List of the complexes of species detected by teleo barcode.**

Due to a lack of taxonomic resolution some species could not be distinguished by the teleo01 barcode:

- “Complex 1”: *Telestes souffia* (Risso, 1827), *Chondrostoma nasus* (L., 1758) and *Parachondrostoma toxostoma* (Vallot, 1837),
- “Complex 3”: *Abramis brama* (L., 1758) and *Blicca bjoerkna* (L., 1758)
- “Complexe 4”: *Alburnus alburnus* (L., 1758) and *Scardinius erythrophthalmus* (L., 1758).
