# Supplementary material for: Spatio-temporal variability of eDNA signal and its implication for fish monitoring in lakes
Source: PLoS One. 2022 Aug 12;17(8):e0272660. doi: 10.1371/journal.pone.0272660 (PMC9374266; doi:10.1371/journal.pone.0272660)

S3. Fig. A. Vertical profiles of the lake Serre-Ponçon. Thermal vertical distribution in Serre-Ponçon for all campaigns.

and Etang des Aulnes

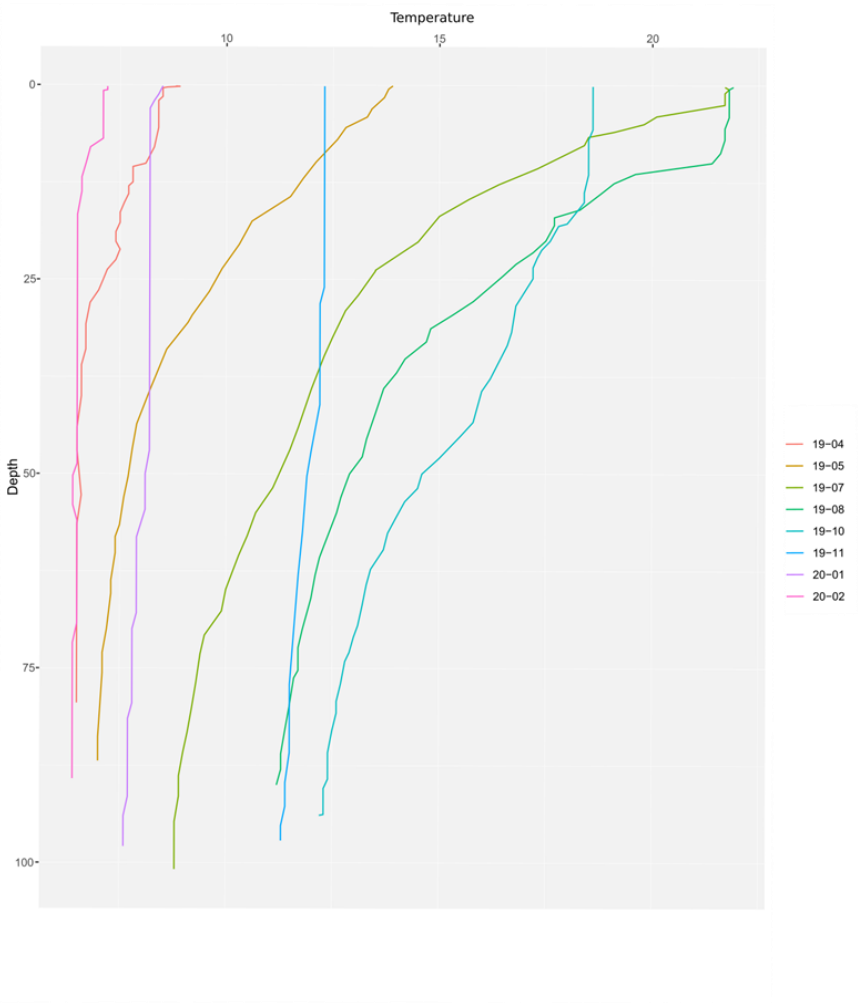

S3. Fig. B. Vertical profiles of the lake Etang des Aulnes. Thermal vertical profile of Etang des Aulnes for all campaigns.

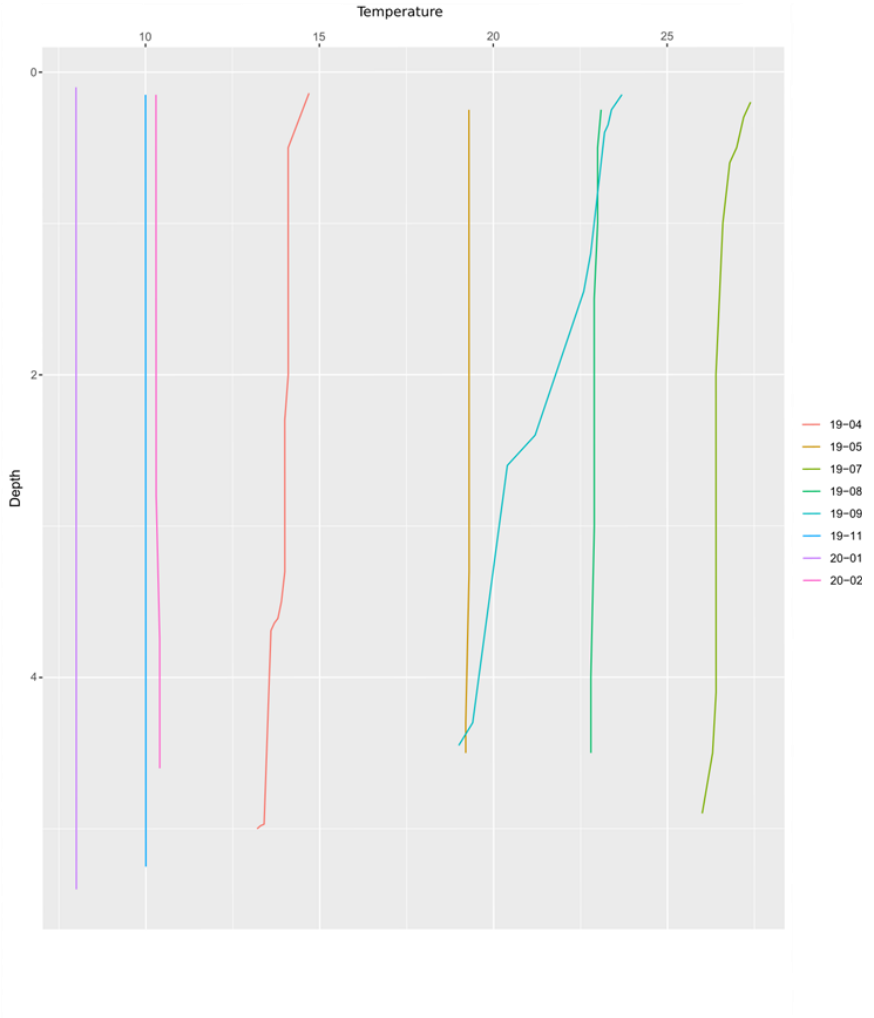

S3. Fig. C. Vertical profiles of the lake Aiguebelette. Thermal vertical profile of Lake Aiguebelette for all campaigns.

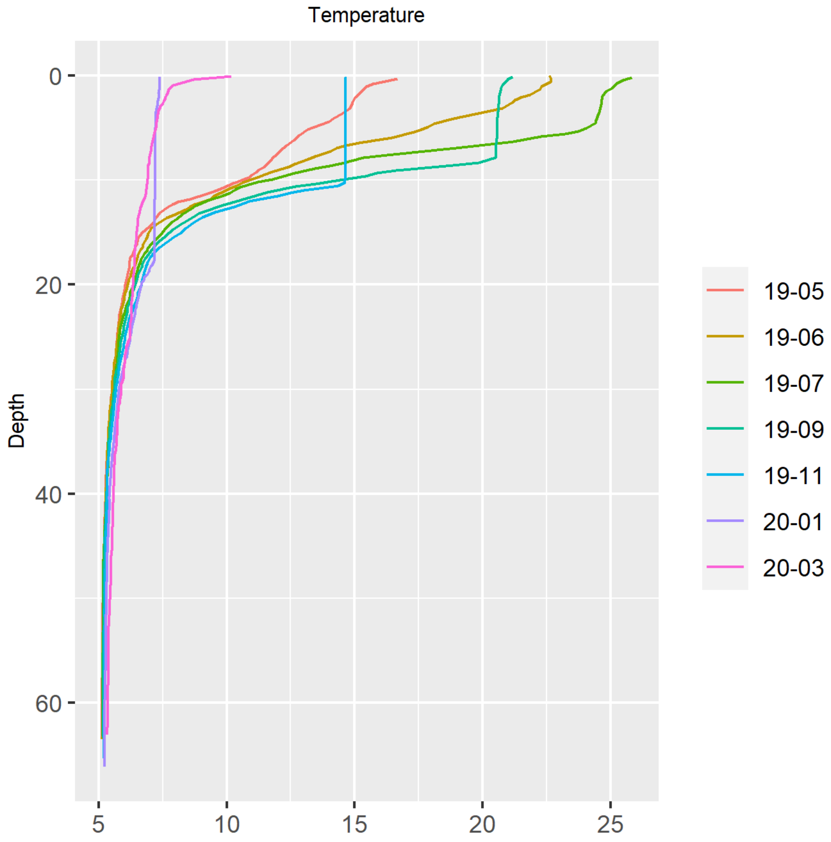

Supplement: S1 Fig — (PDF) [file pone.0272660.s003.pdf]
