# Supplementary material for: Spatio-temporal variability of eDNA signal and its implication for fish monitoring in lakes
Source: PLoS One. 2022 Aug 12;17(8):e0272660. doi: 10.1371/journal.pone.0272660 (PMC9374266; doi:10.1371/journal.pone.0272660)

S4. Fig. NMDS based on the standardised reads. NMDS (gower distance based matrices) with the standardised number of reads for all samples (All), for lake Aiguebelette, for lake Serre-Ponçon and for lake Etang des Aulnes.

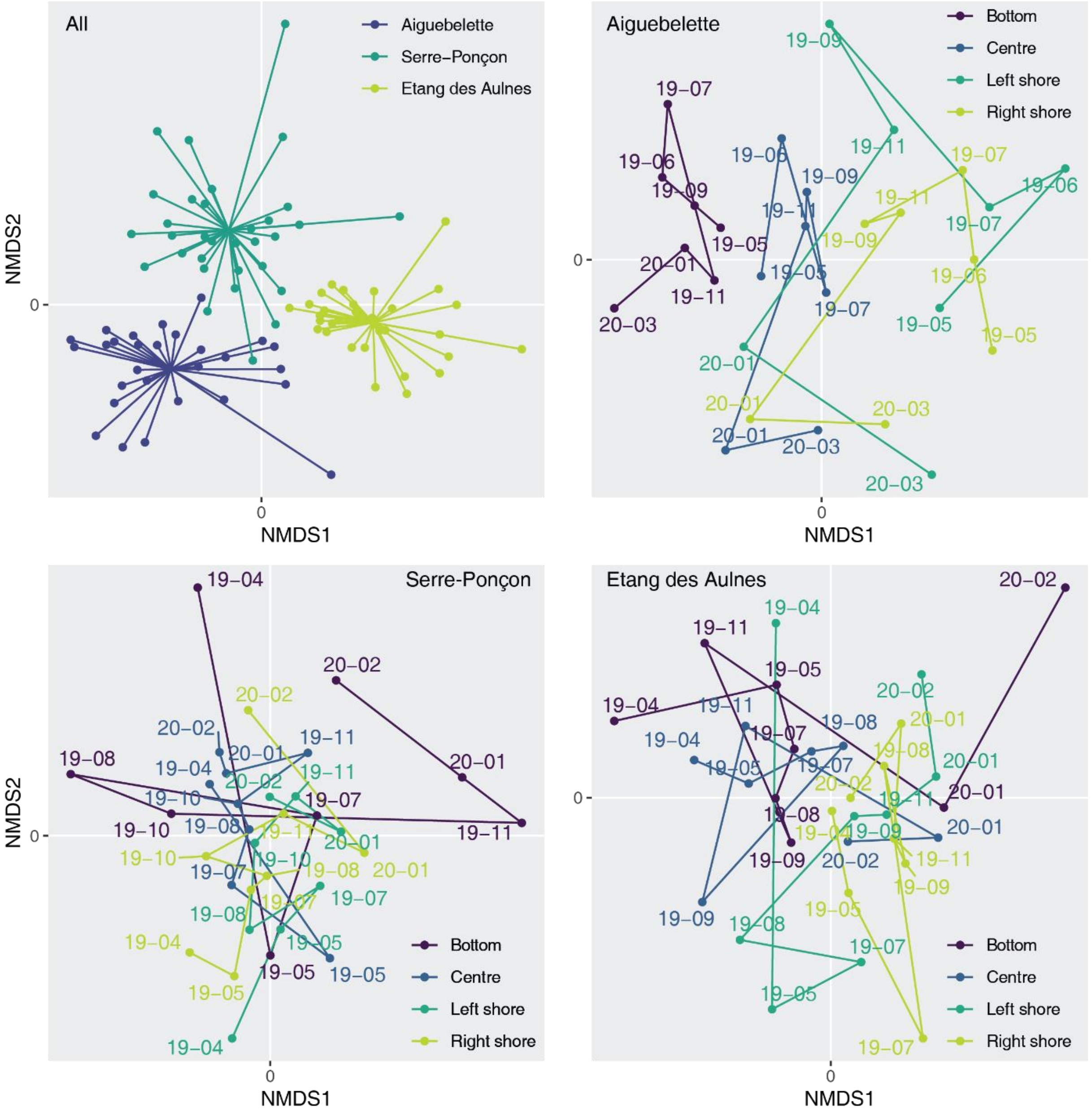

Supplement: S2 Fig — (PDF) [file pone.0272660.s004.pdf]
