# Supplementary material for: Spatio-temporal variability of eDNA signal and its implication for fish monitoring in lakes
Source: PLoS One. 2022 Aug 12;17(8):e0272660. doi: 10.1371/journal.pone.0272660 (PMC9374266; doi:10.1371/journal.pone.0272660)
Supplement: S3 Fig — (PDF) [file pone.0272660.s005.pdf]

S5. Fig. 1. Spatio-temporal variability of the eDNA signal for each species. For each species, the number of reads (standardised) was represented for each campaign and each sampling location (e.g. left shore) and then the proportion of reads was displayed.

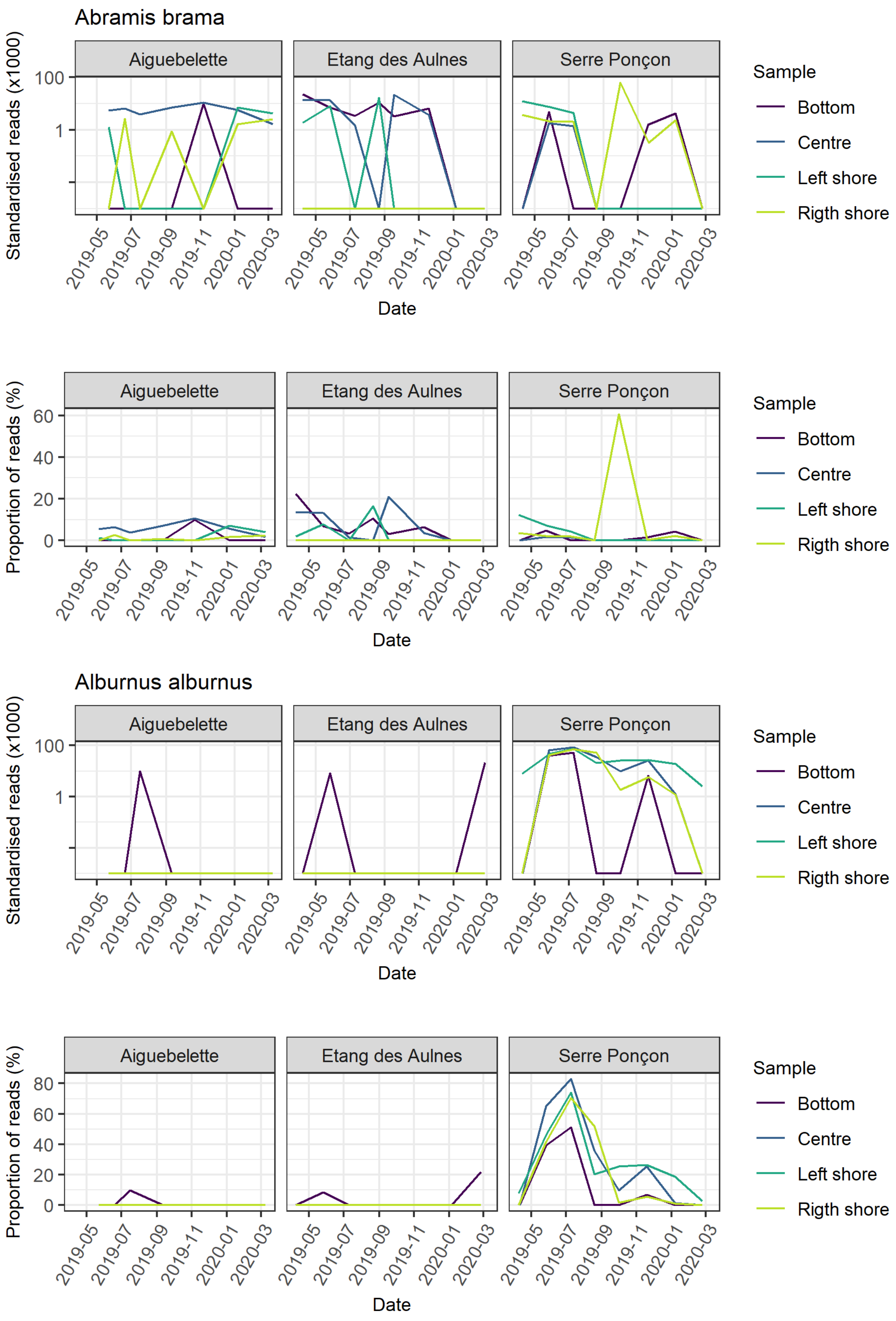

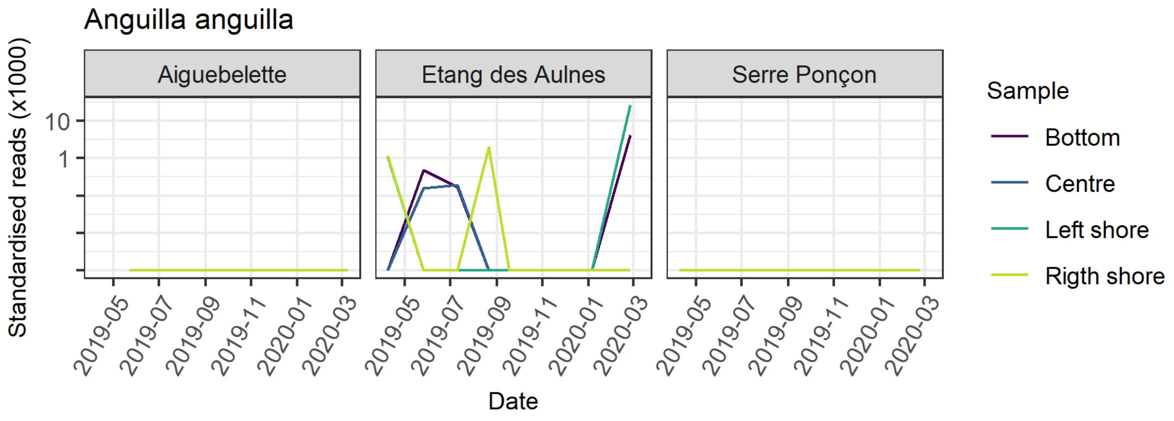
Anguilla anguilla
Standardised reads (x1000)
Aiguebelette
Etang des Aulnes
Serre Ponçon
10
1
2019-05
2019-07
2019-09
2019-11
2020-01
2020-03
Date
Sample
Bottom
Centre
Left shore
Rigth shore

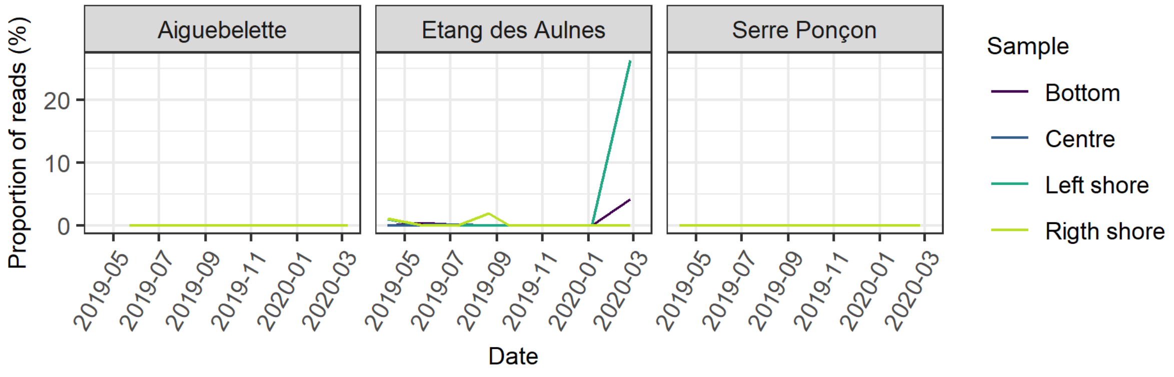
Proportion of reads (%)
Aiguebelette
Etang des Aulnes
Serre Ponçon
20
10
0
2019-05
2019-07
2019-09
2019-11
2020-01
2020-03
Date
Sample
Bottom
Centre
Left shore
Rigth shore

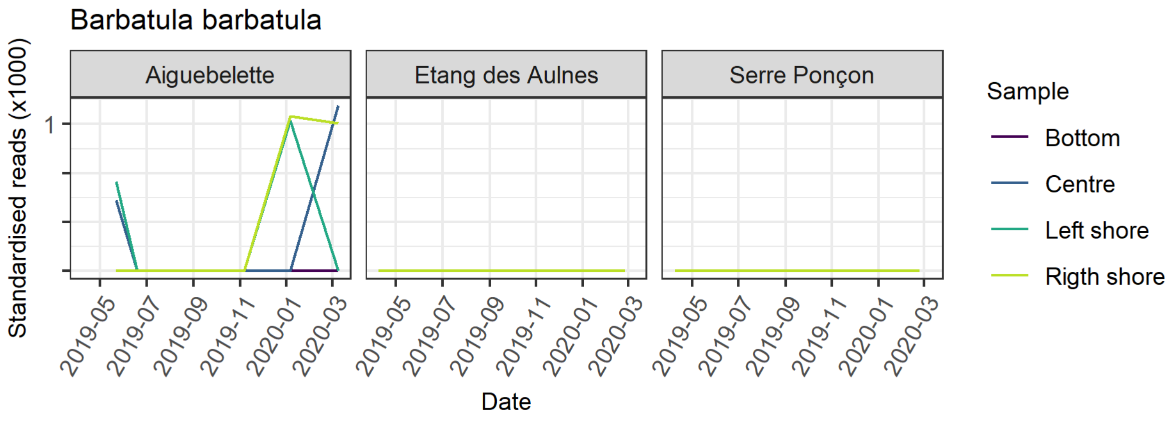
Barbatula barbatula
Standardised reads (x1000)
Aiguebelette
Etang des Aulnes
Serre Ponçon
1
2019-05
2019-07
2019-09
2019-11
2020-01
2020-03
Date
Sample
Bottom
Centre
Left shore
Rigth shore

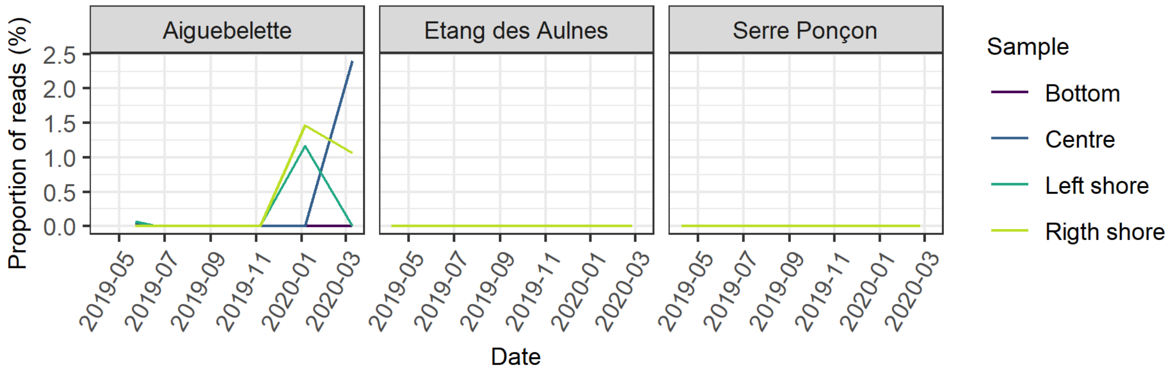
Proportion of reads (%)
Aiguebelette
Etang des Aulnes
Serre Ponçon
2.5
2.0
1.5
1.0
0.5
0.0
2019-05
2019-07
2019-09
2019-11
2020-01
2020-03
Date
Sample
Bottom
Centre
Left shore
Rigth shore

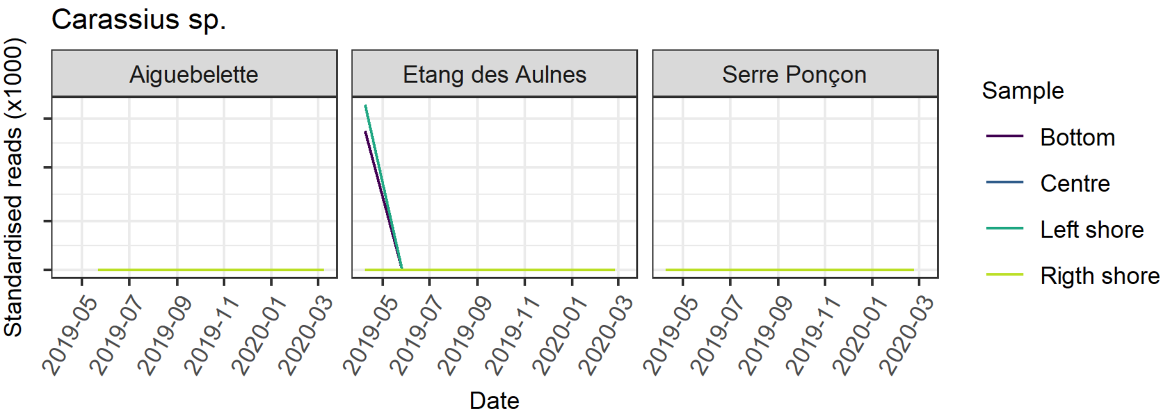

Carassius sp.
Standardised reads (x1000)
Aiguebelette
Etang des Aulnes
Serre Ponçon
2019-05
2019-07
2019-09
2019-11
2020-01
2020-03
Date
Sample
Bottom
Centre
Left shore
Rigth shore

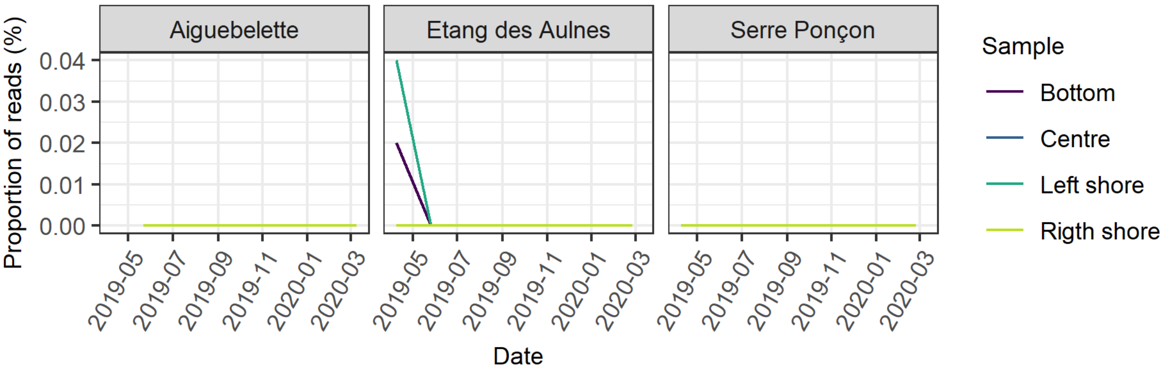

Proportion of reads (%)
0.04
0.03
0.02
0.01
0.00
Aiguebelette
Etang des Aulnes
Serre Ponçon
2019-05
2019-07
2019-09
2019-11
2020-01
2020-03
Date
Sample
Bottom
Centre
Left shore
Rigth shore

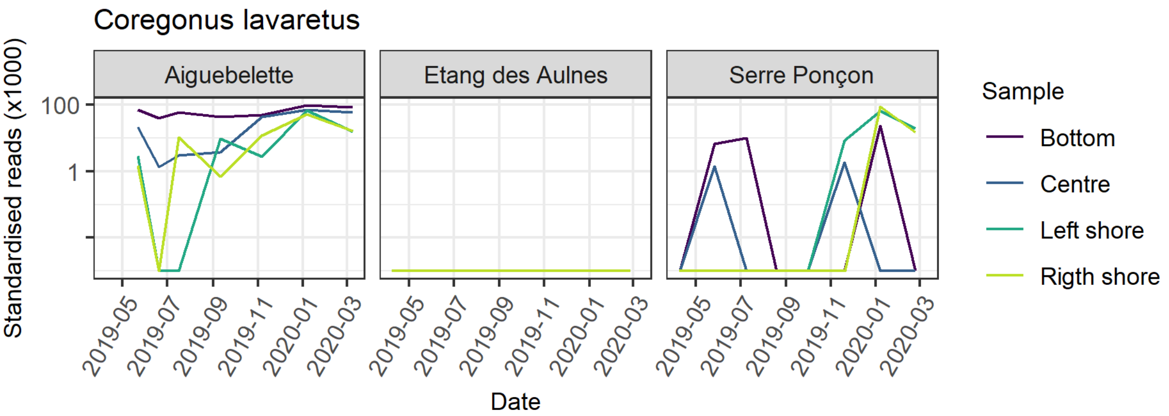

Coregonus lavaretus
Standardised reads (x1000)
100
1
Aiguebelette
Etang des Aulnes
Serre Ponçon
2019-05
2019-07
2019-09
2019-11
2020-01
2020-03
Date
Sample
Bottom
Centre
Left shore
Rigth shore

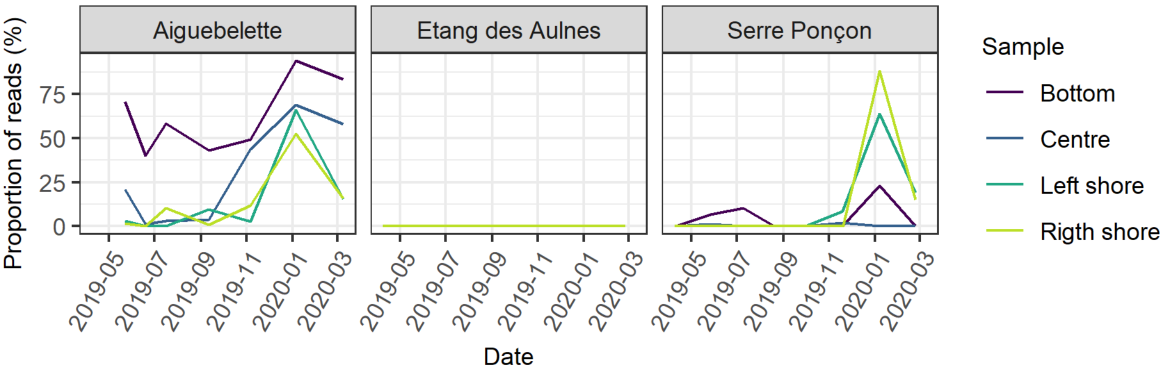

Proportion of reads (%)
75
50
25
0
Aiguebelette
Etang des Aulnes
Serre Ponçon
2019-05
2019-07
2019-09
2019-11
2020-01
2020-03
Date
Sample
Bottom
Centre
Left shore
Rigth shore

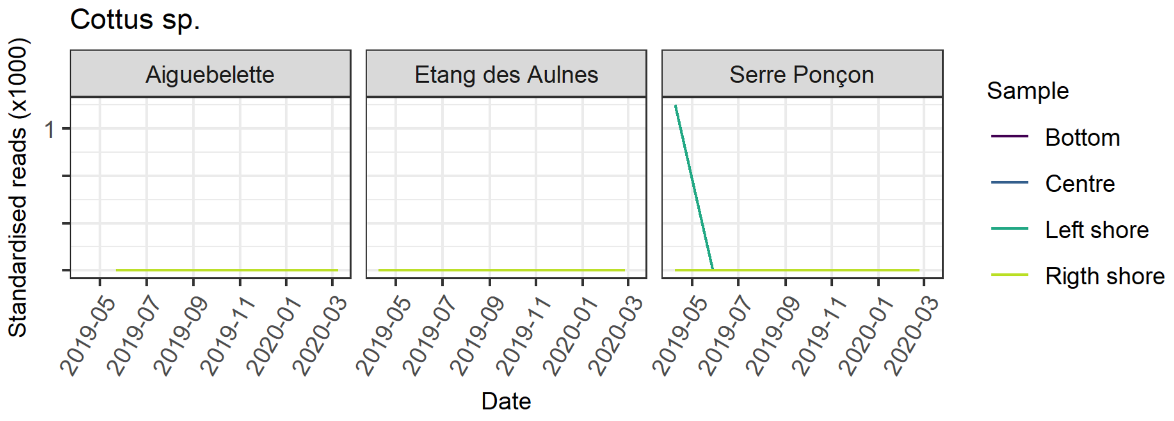
Cottus sp.
Aiguebelette
Etang des Aulnes
Serre Ponçon
Standardised reads (x1000)
1
2019-05
2019-07
2019-09
2019-11
2020-01
2020-03
Date
Sample
Bottom
Centre
Left shore
Rigth shore

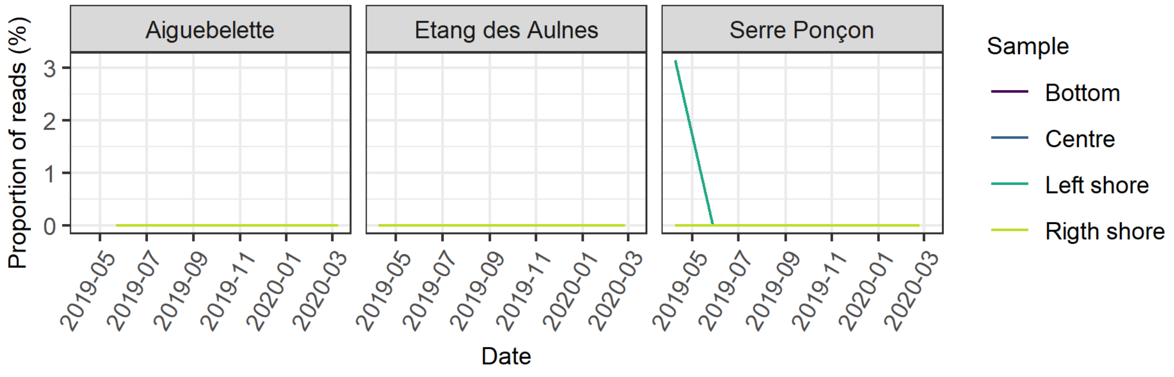
Aiguebelette
Etang des Aulnes
Serre Ponçon
Proportion of reads (%)
3
2
1
0
2019-05
2019-07
2019-09
2019-11
2020-01
2020-03
Date
Sample
Bottom
Centre
Left shore
Rigth shore

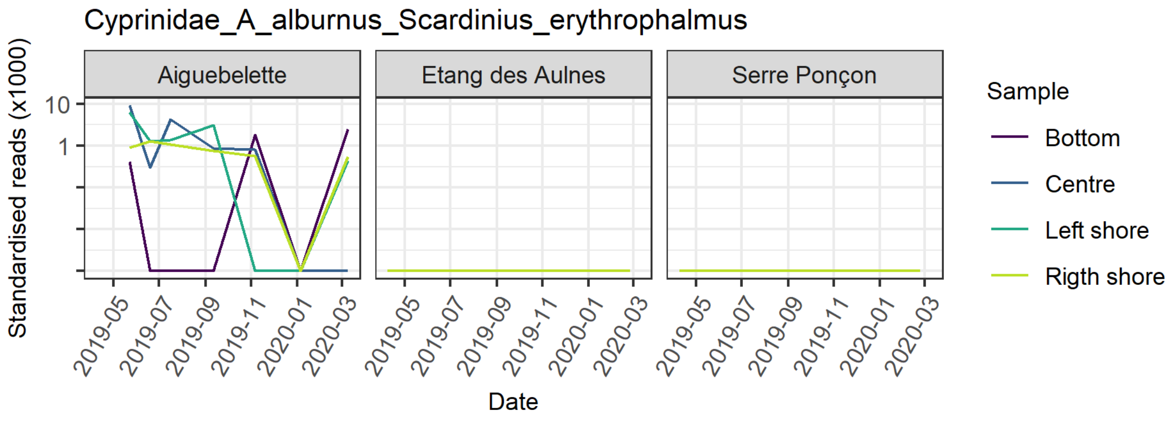
Cyprinidae_A_alburnus_Scardinius_erythrophalmus
Aiguebelette
Etang des Aulnes
Serre Ponçon
Standardised reads (x1000)
10
1
2019-05
2019-07
2019-09
2019-11
2020-01
2020-03
Date
Sample
Bottom
Centre
Left shore
Rigth shore

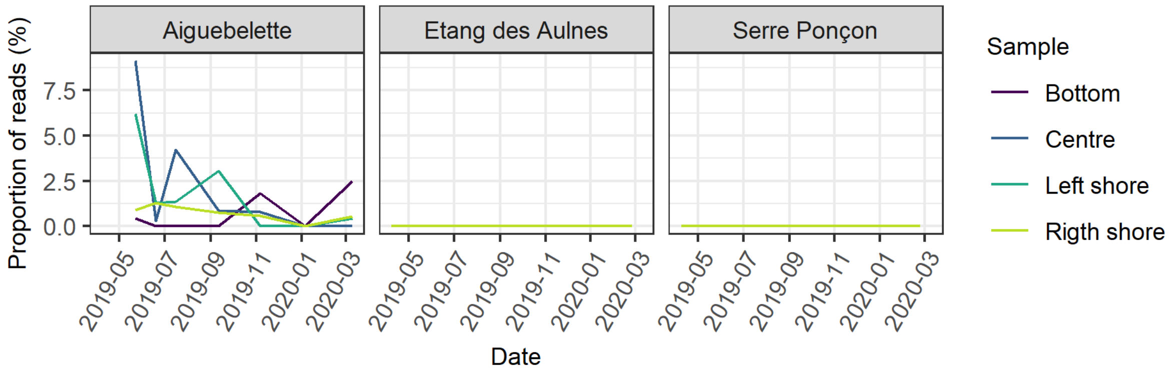
Aiguebelette
Etang des Aulnes
Serre Ponçon
Proportion of reads (%)
7.5
5.0
2.5
0.0
2019-05
2019-07
2019-09
2019-11
2020-01
2020-03
Date
Sample
Bottom
Centre
Left shore
Rigth shore

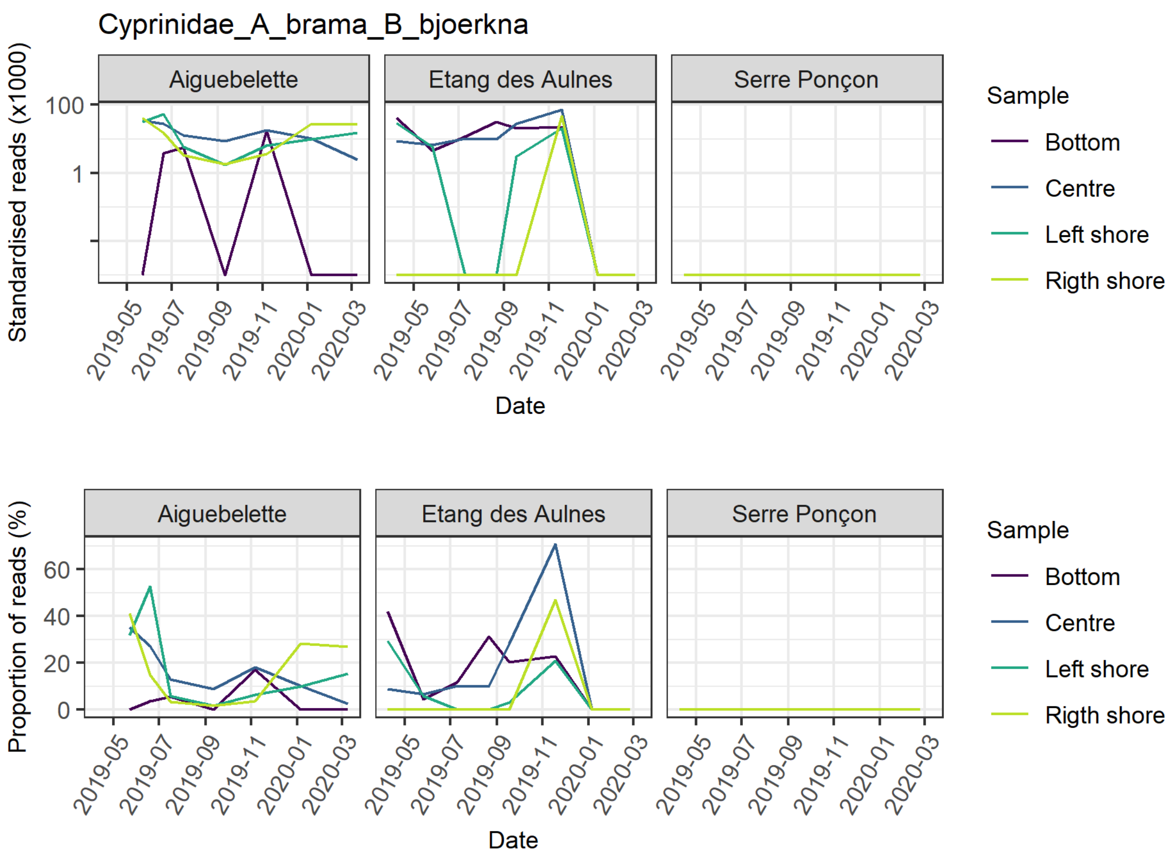
Cyprinidae_A_brama_B_bjoerkna
Standardised reads (x1000)
100
1
Aiguebelette
Etang des Aulnes
Serre Ponçon
2019-05
2019-07
2019-09
2019-11
2020-01
2020-03
Date
Sample
Bottom
Centre
Left shore
Rigth shore
Proportion of reads (%)
60
40
20
0
Aiguebelette
Etang des Aulnes
Serre Ponçon
Date
Sample
Bottom
Centre
Left shore
Rigth shore

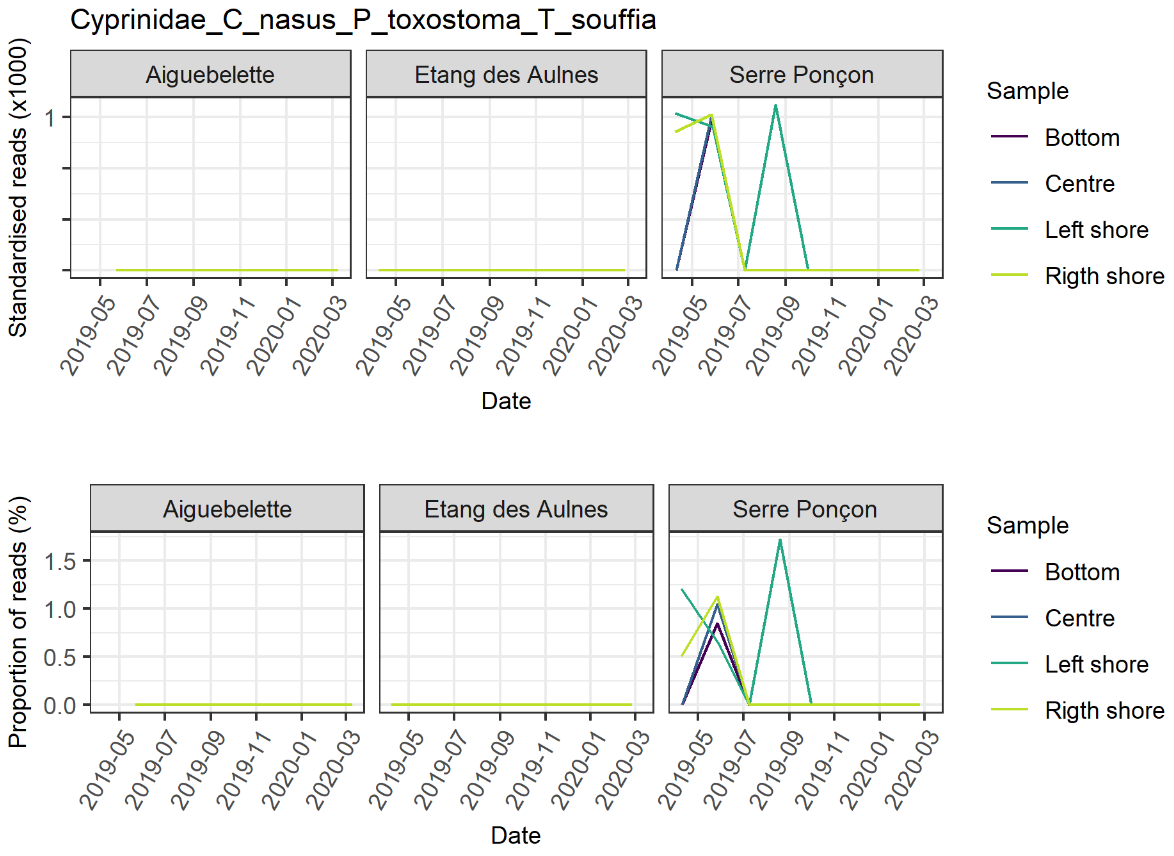
Cyprinidae_C_nasus_P_toxostoma_T_souffia
Standardised reads (x1000)
1
Aiguebelette
Etang des Aulnes
Serre Ponçon
2019-05
2019-07
2019-09
2019-11
2020-01
2020-03
Date
Sample
Bottom
Centre
Left shore
Rigth shore
Proportion of reads (%)
1.5
1.0
0.5
0.0
Aiguebelette
Etang des Aulnes
Serre Ponçon
Date
Sample
Bottom
Centre
Left shore
Rigth shore

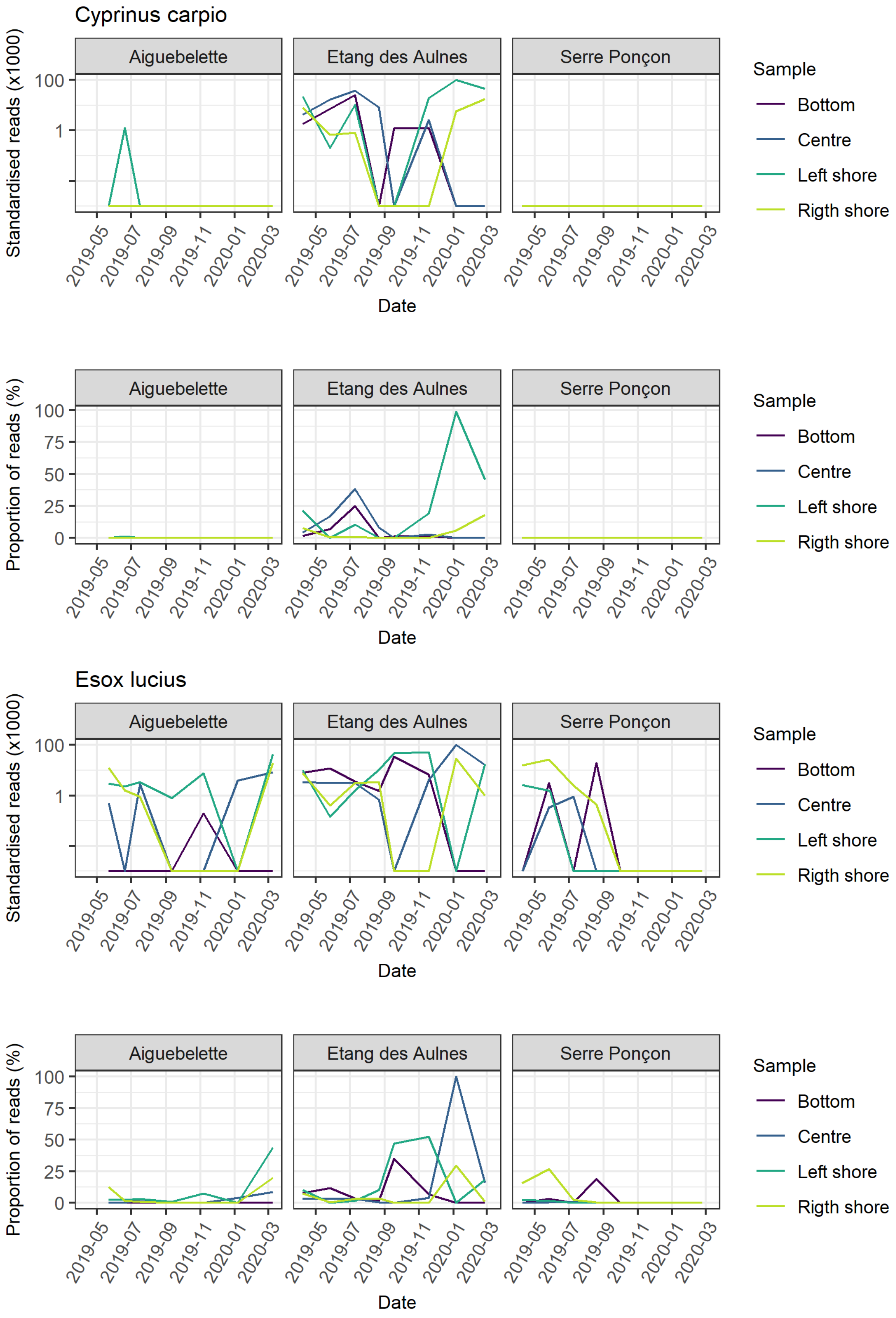
Cyprinus carpio
Esox lucius
Aiguebelette
Etang des Aulnes
Serre Ponçon
Standardised reads (x1000)
Proportion of reads (%)
100
1
75
50
25
0
2019-05
2019-07
2019-09
2019-11
2020-01
2020-03
Date
Sample
Bottom
Centre
Left shore
Rigth shore

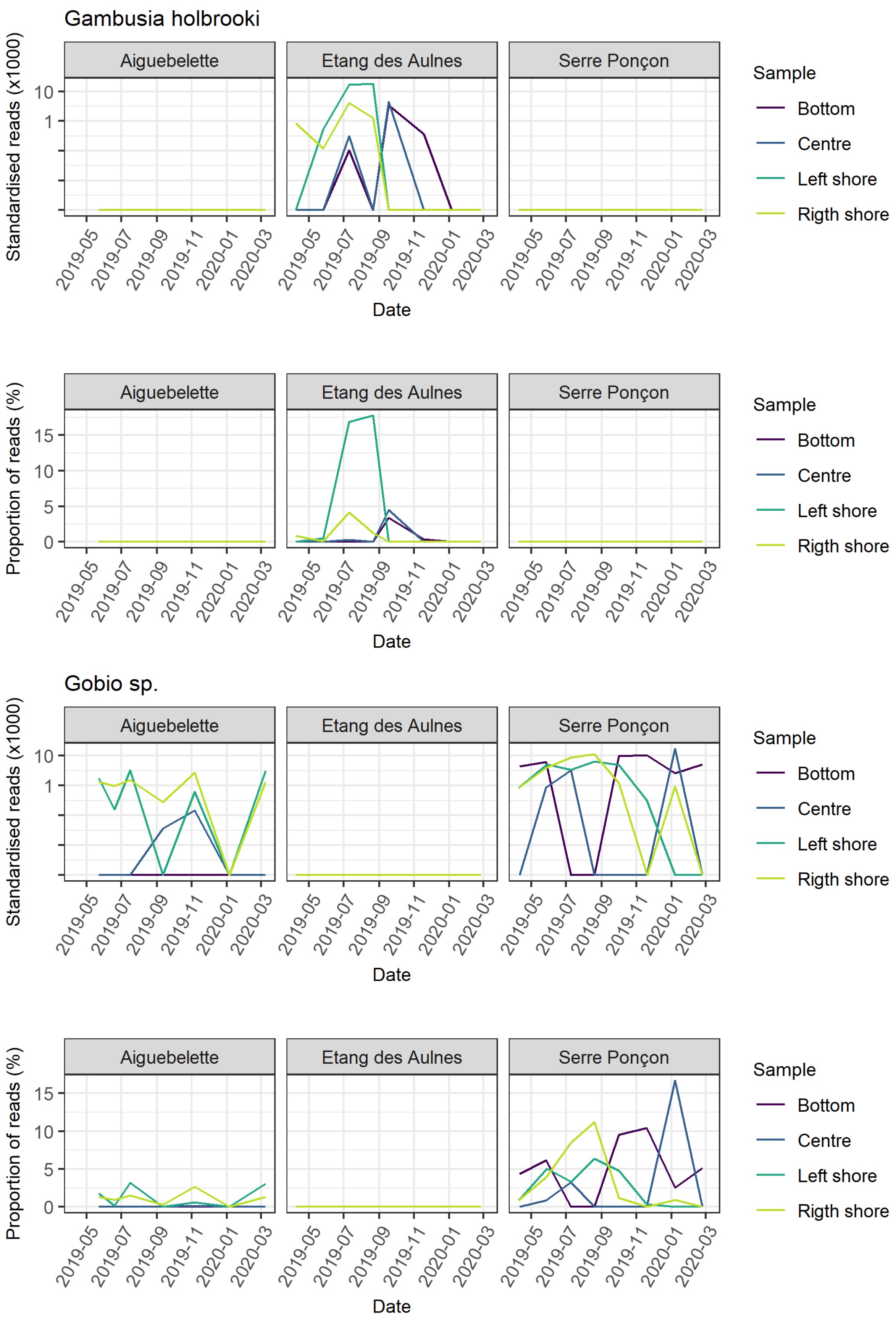
Gambusia holbrooki
Standardised reads (x1000)
Proportion of reads (%)
Gobio sp.
Aiguebelette
Etang des Aulnes
Serre Ponçon
Date
Sample
Bottom
Centre
Left shore
Rigth shore

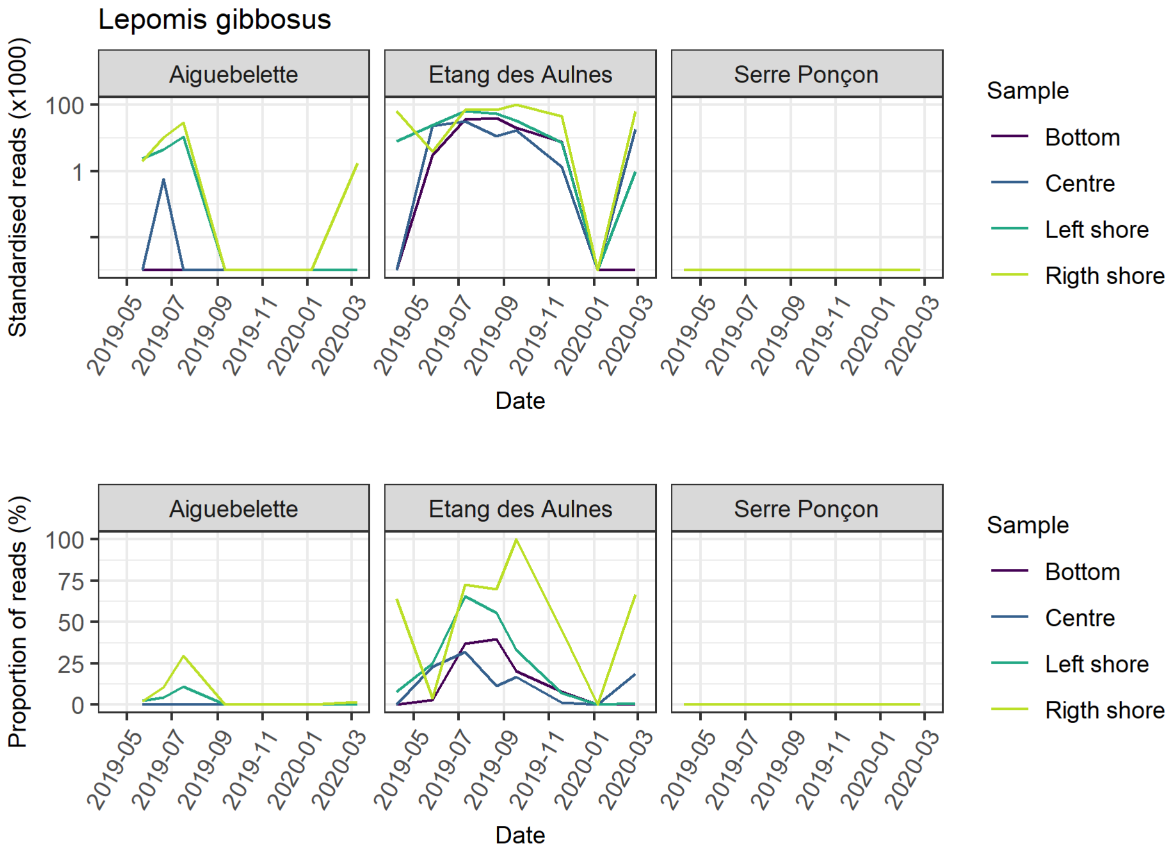
Lepomis gibbosus
Standardised reads (x1000)
Proportion of reads (%)
Aiguebelette
Etang des Aulnes
Serre Ponçon
Date
Sample
Bottom
Centre
Left shore
Rigth shore

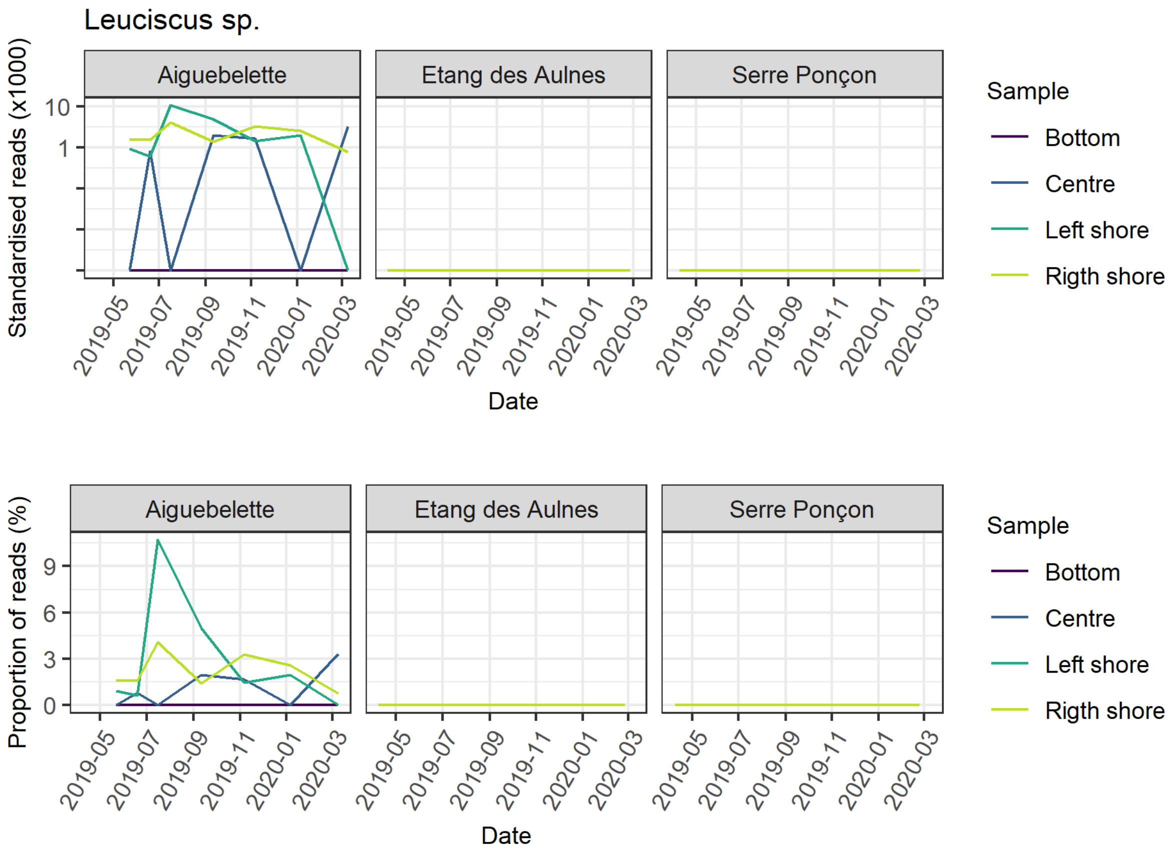
Leuciscus sp.
Standardised reads (x1000)
Proportion of reads (%)
Aiguebelette
Etang des Aulnes
Serre Ponçon
Date
Sample
Bottom
Centre
Left shore
Rigth shore

Lota lota

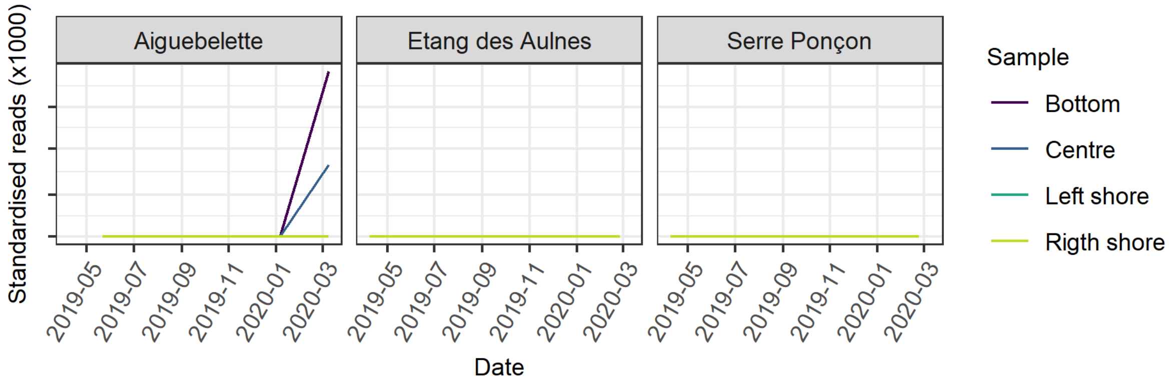

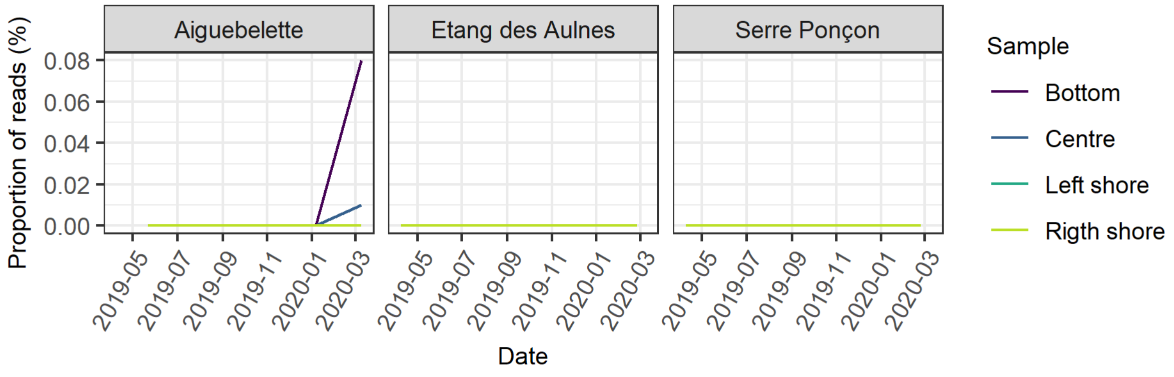

Oncorhynchus mykiss

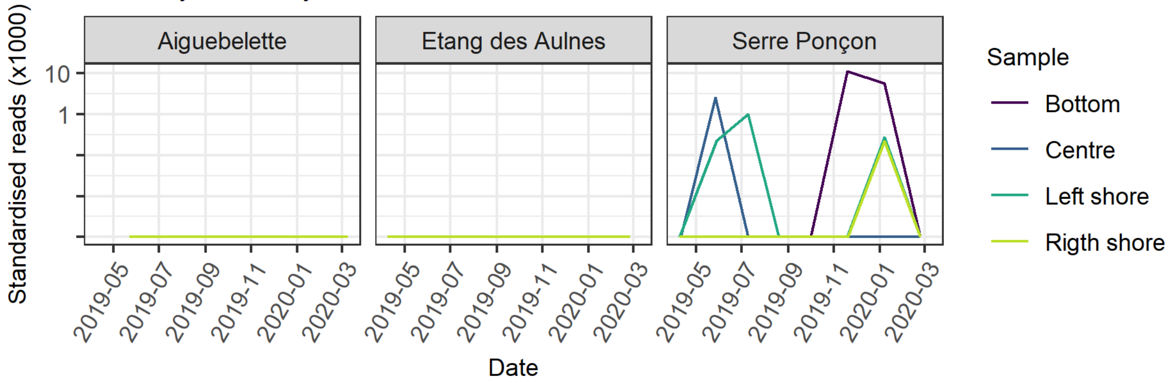

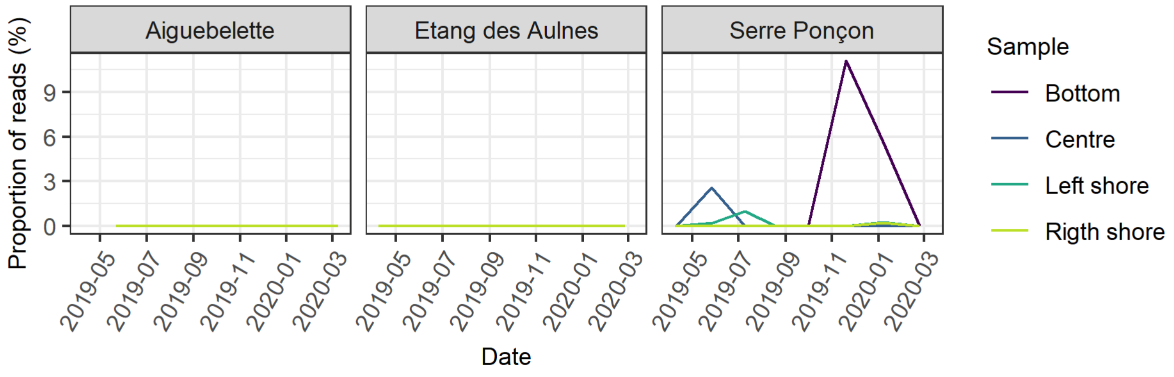

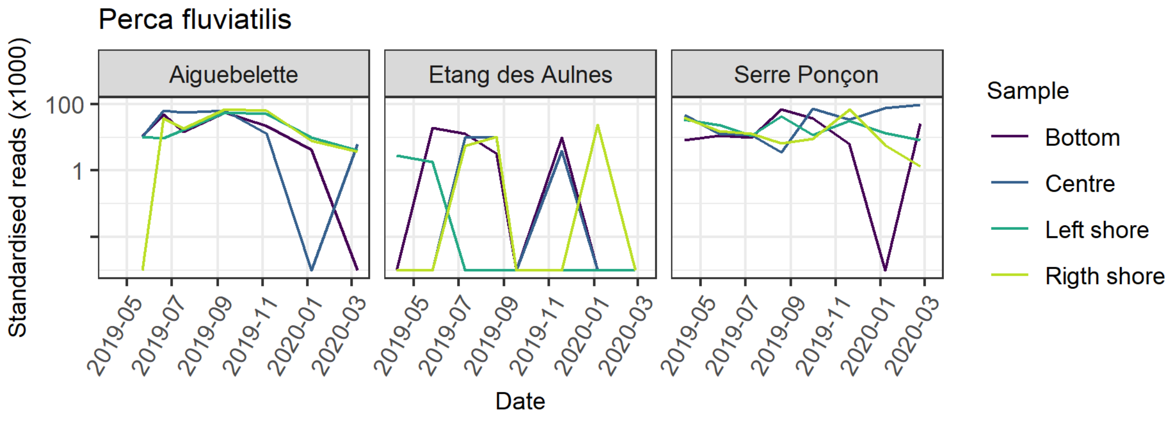
Perca fluviatilis
Standardised reads (x1000)
100
1
Aiguebelette
Etang des Aulnes
Serre Ponçon
2019-05
2019-07
2019-09
2019-11
2020-01
2020-03
Date
Sample
Bottom
Centre
Left shore
Rigth shore

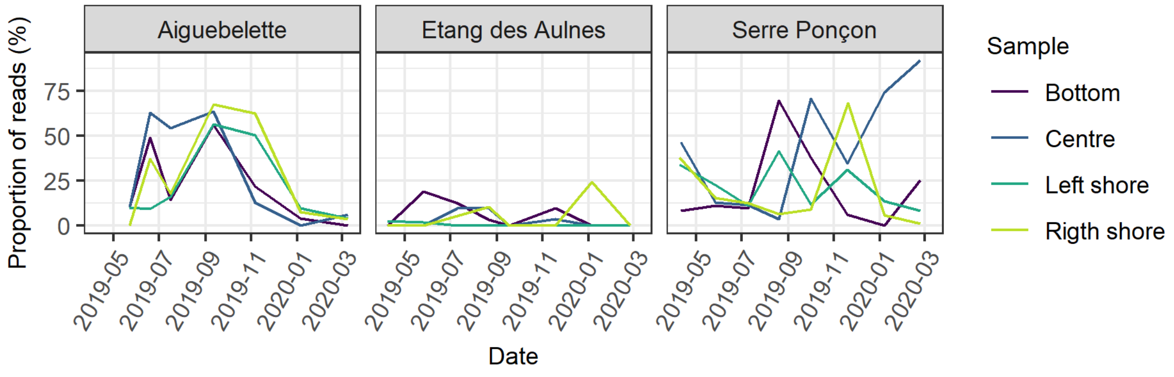
Proportion of reads (%)
75
50
25
0
Aiguebelette
Etang des Aulnes
Serre Ponçon
2019-05
2019-07
2019-09
2019-11
2020-01
2020-03
Date
Sample
Bottom
Centre
Left shore
Rigth shore

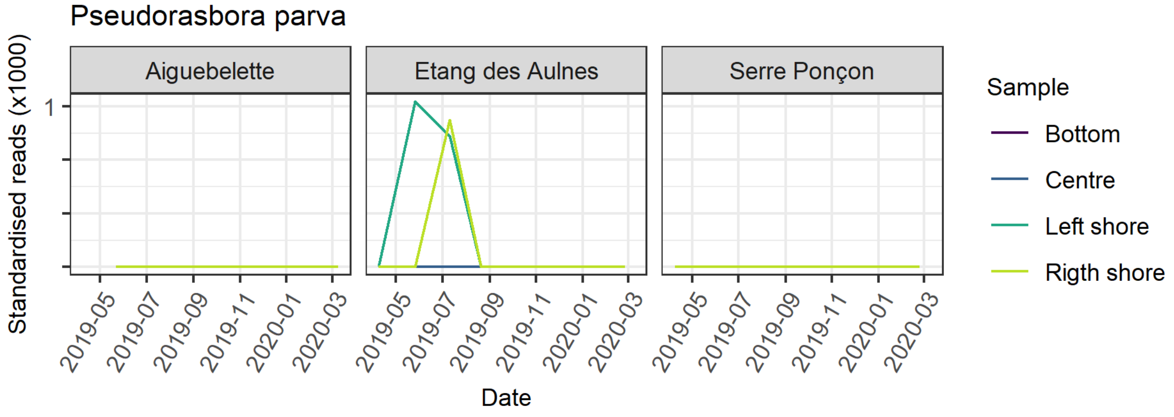
Pseudorasbora parva
Standardised reads (x1000)
1
Aiguebelette
Etang des Aulnes
Serre Ponçon
2019-05
2019-07
2019-09
2019-11
2020-01
2020-03
Date
Sample
Bottom
Centre
Left shore
Rigth shore

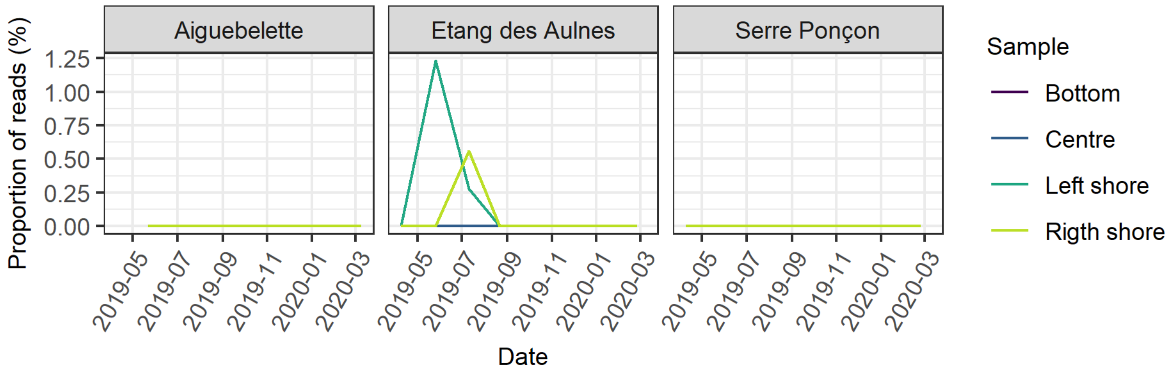
Proportion of reads (%)
1.25
1.00
0.75
0.50
0.25
0.00
Aiguebelette
Etang des Aulnes
Serre Ponçon
2019-05
2019-07
2019-09
2019-11
2020-01
2020-03
Date
Sample
Bottom
Centre
Left shore
Rigth shore

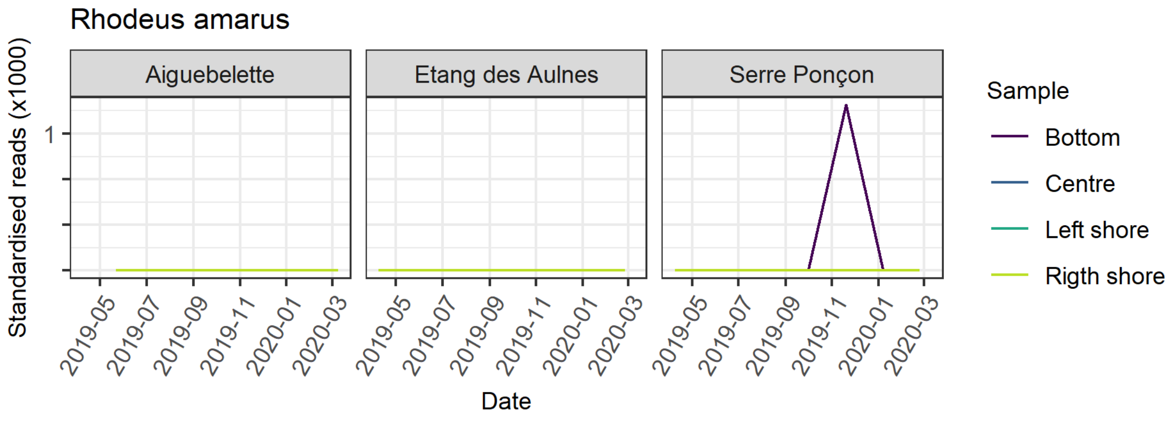
Rhodeus amarus
Standardised reads (x1000)
1
Aiguebelette
Etang des Aulnes
Serre Ponçon
2019-05
2019-07
2019-09
2019-11
2020-01
2020-03
Date
Sample
Bottom
Centre
Left shore
Rigth shore

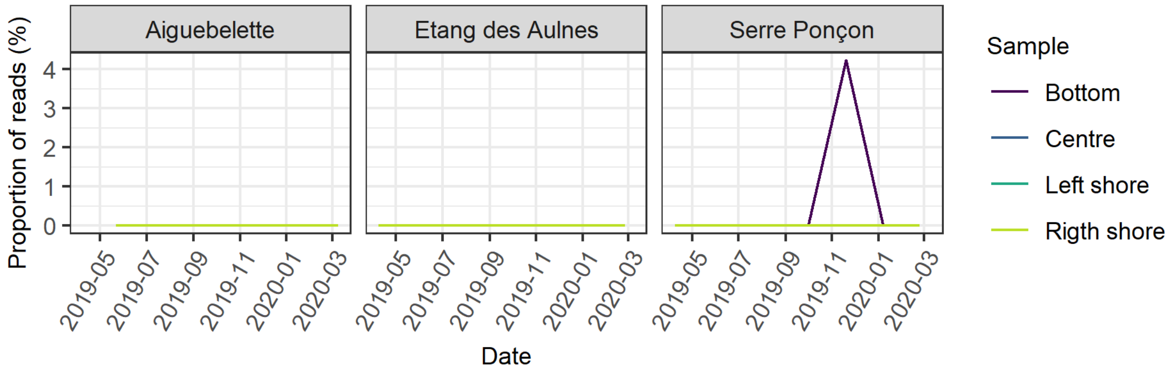
Proportion of reads (%)
0
1
2
3
4
Aiguebelette
Etang des Aulnes
Serre Ponçon
2019-05
2019-07
2019-09
2019-11
2020-01
2020-03
Date
Sample
Bottom
Centre
Left shore
Rigth shore

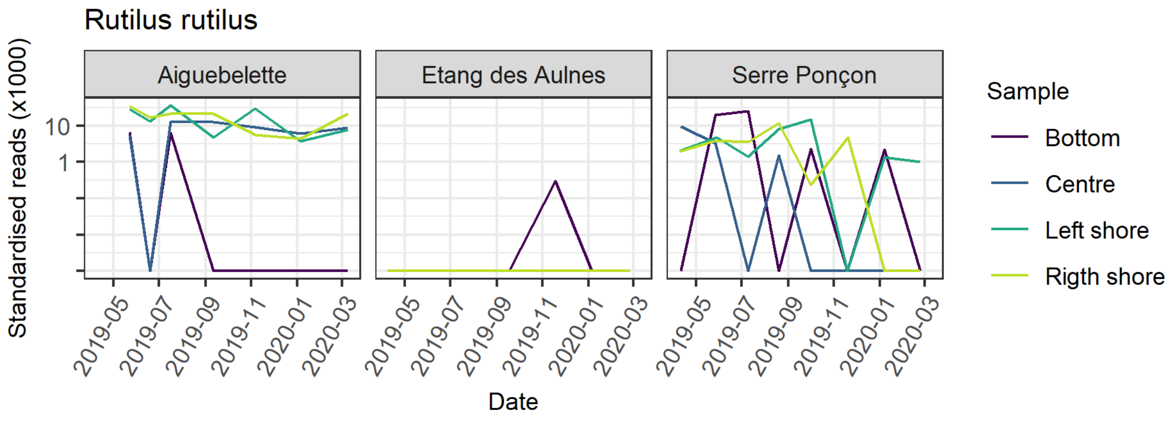
Rutilus rutilus
Standardised reads (x1000)
1
10
Aiguebelette
Etang des Aulnes
Serre Ponçon
2019-05
2019-07
2019-09
2019-11
2020-01
2020-03
Date
Sample
Bottom
Centre
Left shore
Rigth shore

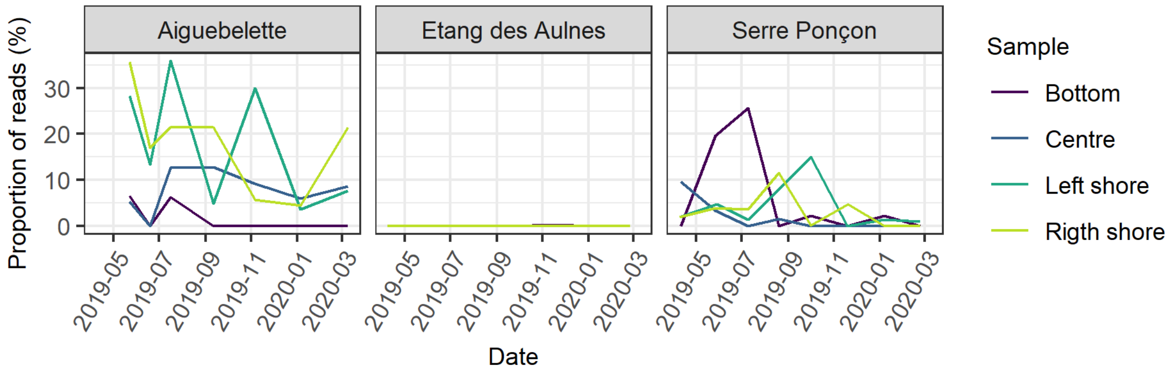
Proportion of reads (%)
0
10
20
30
Aiguebelette
Etang des Aulnes
Serre Ponçon
2019-05
2019-07
2019-09
2019-11
2020-01
2020-03
Date
Sample
Bottom
Centre
Left shore
Rigth shore

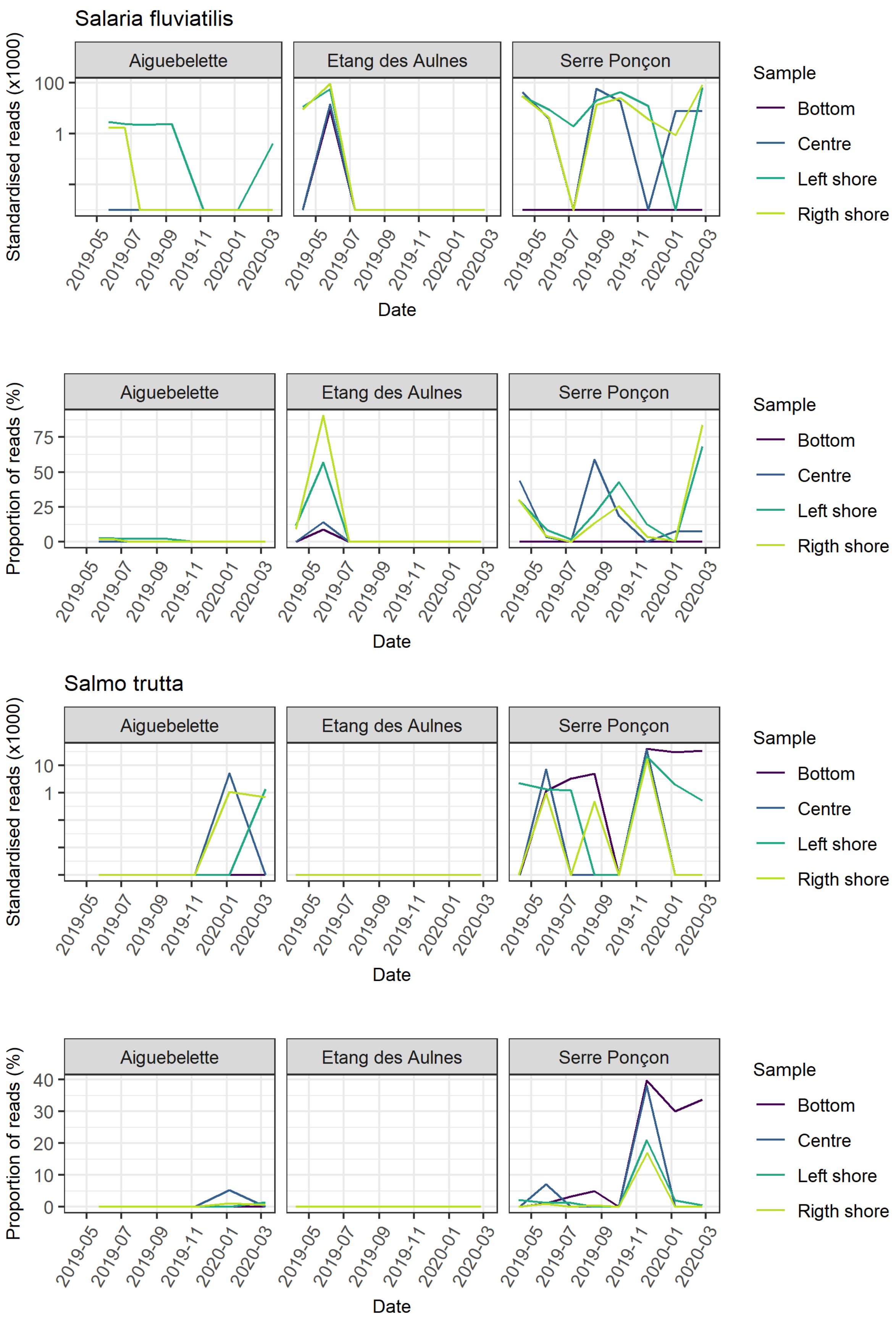

Salaria fluviatilis
Aiguebelette
Etang des Aulnes
Serre Ponçon
Standardised reads (x1000)
100
1
2019-05
2019-07
2019-09
2019-11
2020-01
2020-03
Date
Sample
Bottom
Centre
Left shore
Rigth shore
Proportion of reads (%)
75
50
25
0
Salmo trutta
10
1
40
30
20
10
0

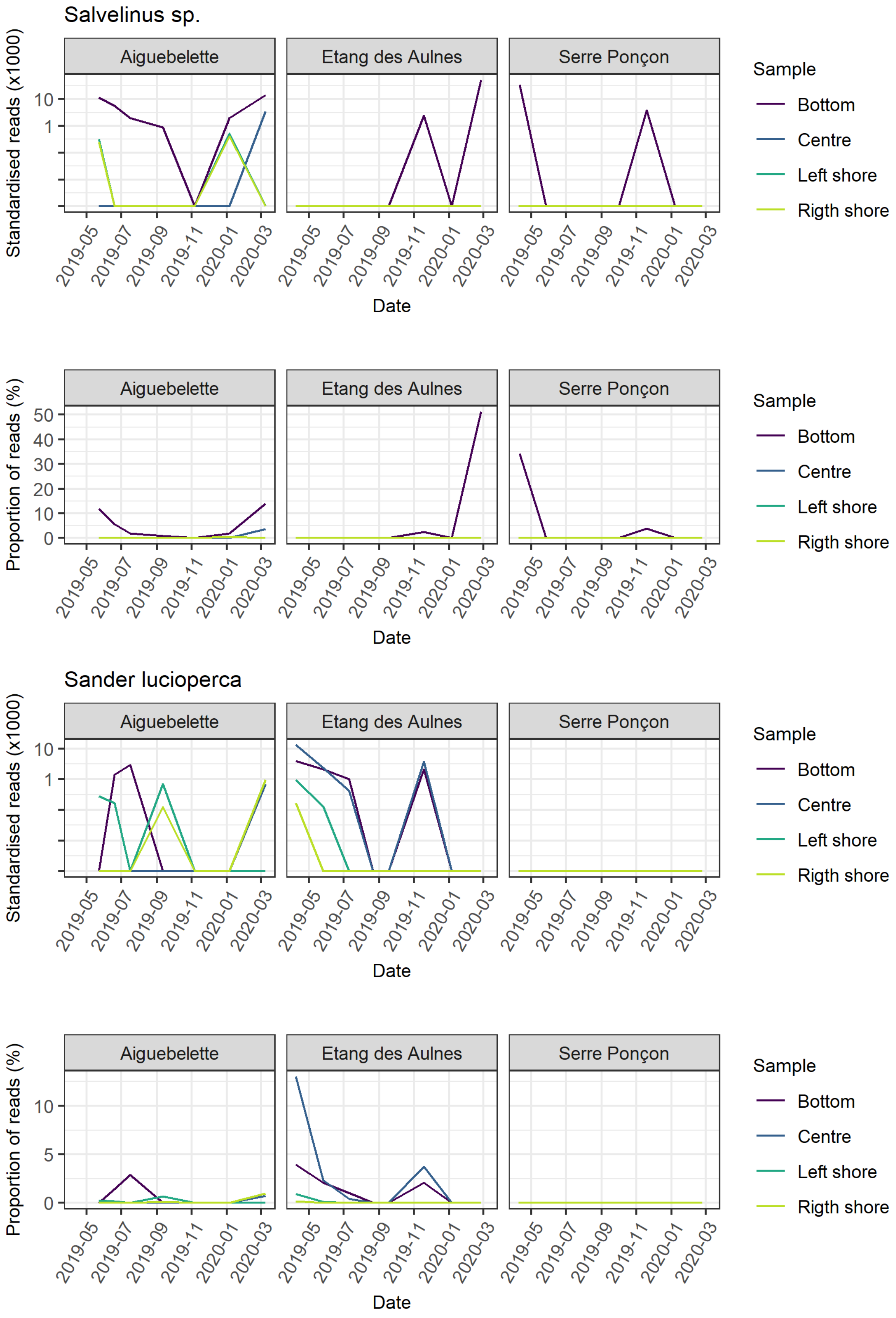
Salvelinus sp.
Standardised reads (x1000)
Proportion of reads (%)
Sander lucioperca
Aiguebelette
Etang des Aulnes
Serre Ponçon
10
1
50
40
30
20
10
5
0
2019-05
2019-07
2019-09
2019-11
2020-01
2020-03
Date
Sample
Bottom
Centre
Left shore
Rigth shore

Scardinius erythrophthalmus

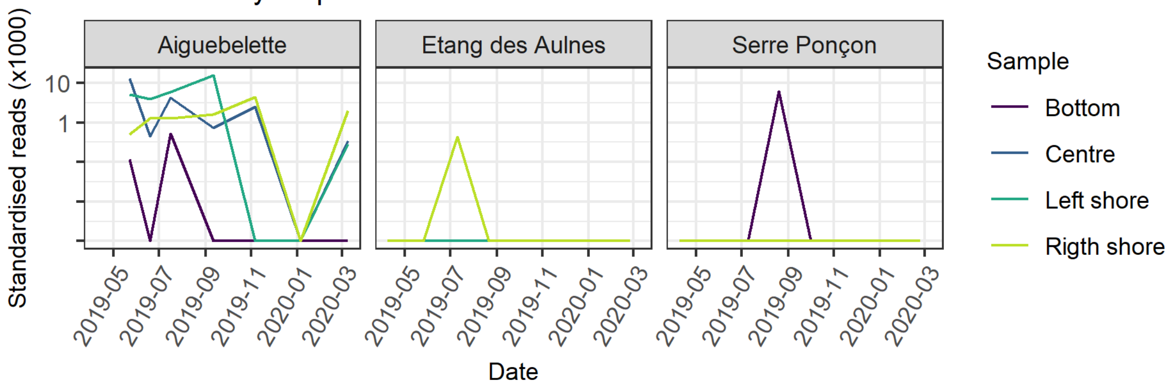

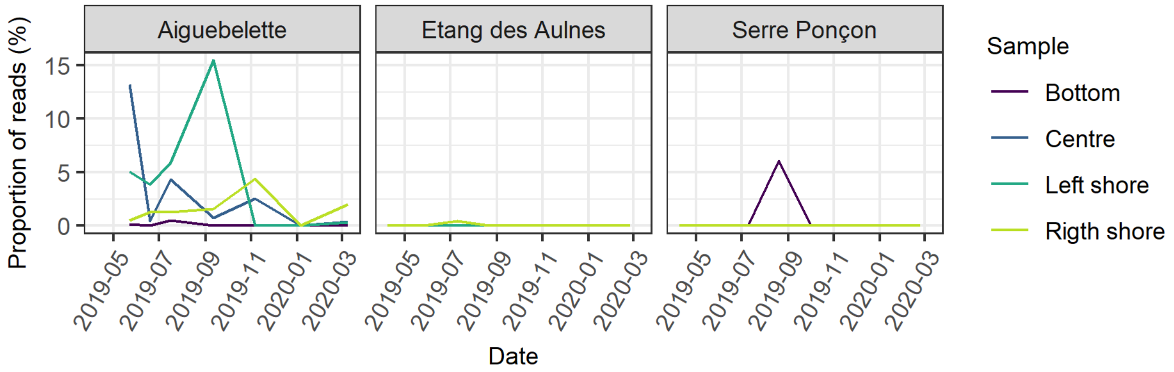

Silurus glanis

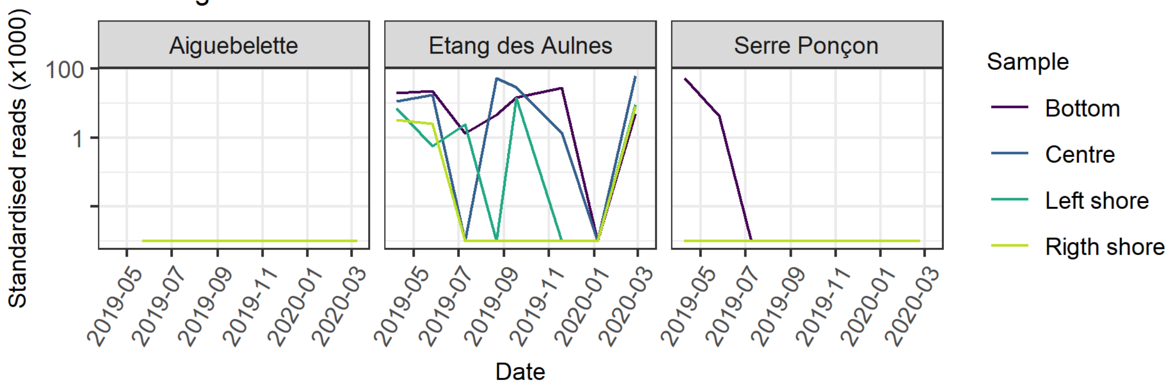

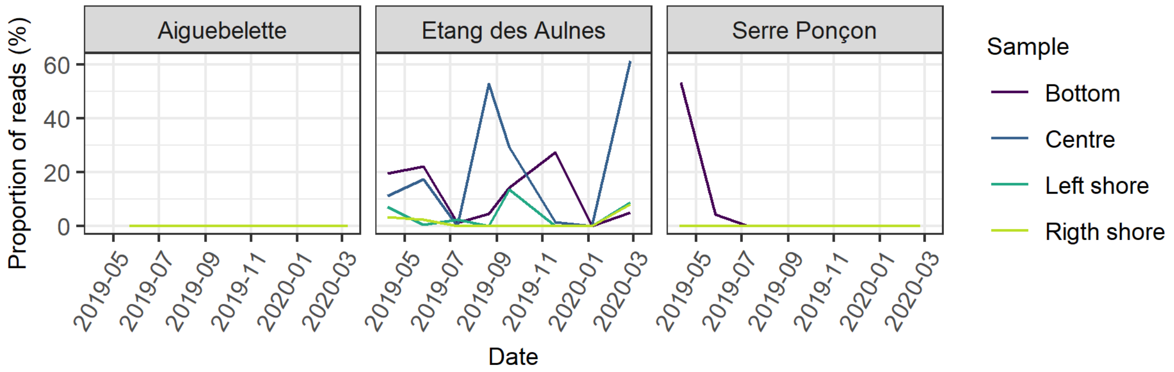

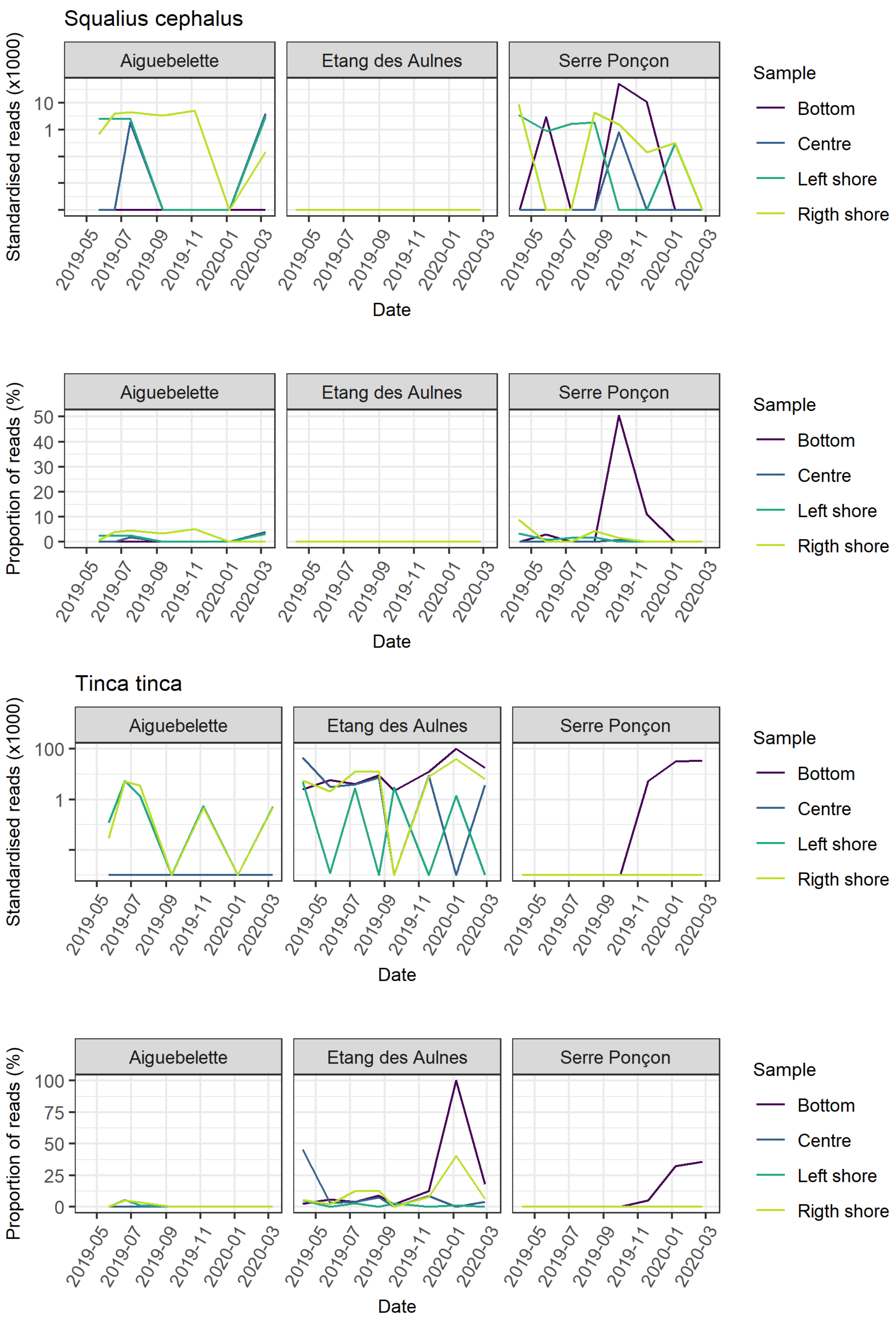
Squalius cephalus
Standardised reads (x1000)
Aiguebelette
Etang des Aulnes
Serre Ponçon
10
1
2019-05
2019-07
2019-09
2019-11
2020-01
2020-03
Date
Sample
Bottom
Centre
Left shore
Rigth shore
Proportion of reads (%)
50
40
30
20
10
0
Tinca tinca
100
75
25
