# Supplementary material for: Spatio-temporal variability of eDNA signal and its implication for fish monitoring in lakes
Source: PLoS One. 2022 Aug 12;17(8):e0272660. doi: 10.1371/journal.pone.0272660 (PMC9374266; doi:10.1371/journal.pone.0272660)
Supplement: S1 Appendix — (PDF) [file pone.0272660.s006.pdf]

S6. Appendix. A. English translation of the IGN answer concerning the compatibility of the licence etalab 2 with the CC BY licence.

Good morning,

There is no counter-indication in the terms of the licence Etalab 2.0 as long as you indicate the origin and the date of the last upgrade of the resources used as it is done in your document.

Regards

S6. Appendix. B. The original answer (in French) received from the IGN.

Logez Maxime

**De:**	contact.geoservices <contact.geoservices@ign.fr>
**Envoyé:**	vendredi 24 juin 2022 09:47
**À:**	Maxime LOGEZ
**Objet:**	RE : Faire état d'une autre difficulté CRM:0107874

Bonjour,

Il n'y a aucune contre-indications dans les termes de la licence Etalab 2.0 , tant que vous indiquez la paternité et la date de dernière mise à jour de les ressources utilisées comme cela est fait sur votre document.

Cordialement,

RÉPUBLIQUE FRANÇAISE
Liberté
Égalité
Fraternité

IGN
INSTITUT NATIONAL DE L'INFORMATION GÉOGRAPHIQUE ET FORESTIÈRE

CHANGER D'ÉCHELLE

Relation Utilisateurs - Géoservices IGN
Courriel : contact.geoservices@ign.fr

Afin de mieux répondre à vos attentes, nous vous invitons à partager votre avis sur notre nouveau site Géoservices

Les modalités d'accès aux services web évoluent au 1er février 2022
Cliquez ici pour en savoir plus

Inscrivez-vous à la lettre Géoservices (accessible en bas de page du site)

------------------- Message d'origine -------------------

De : pgie.geoservices.p <pgie.geoservices.p@agriculture.gouv.fr>;
Reçu : Thu Jun 23 2022 17:47:10 GMT+0200 (heure d'été d'Europe centrale)
À : contact.geoservices <contact.geoservices@ign.fr>;
Sujet : Faire état d'une autre difficulté

géoservices

Informations sur le demandeur :
- Nom : Logez
- Prénom : Maxime
- Organisme : INRAE
- Adresse email : maxime.logez@inrae.fr
- Numéro de téléphone :

Description de la demande :
Bonjour, Je voulais savoir si la licence etalab 2.0 était compatible avec la licence CC BY 4.0. Je souhaite publier un article dans une revue e access (Plos One) qui distribue son contenu sous licence cc BY 4.0. J'ai créer une illustration à partir de données IGN (BD Ortho, voir figure en spécifiant l'origine des données. Est-ce que je peux la diffuser dans ce cadre (CC BY 4.0) ? Bien cordialement, Maxime Logez

Envoyé par Géoservices.ign.fr
Contactez-nous
Institut national de l'information géographique et forestère (IGN)
73 avenue de Paris 94165 SAINT-MANDÉ CEDEX
