# Supplementary material for: Spatio-temporal variability of eDNA signal and its implication for fish monitoring in lakes
Source: PLoS One. 2022 Aug 12;17(8):e0272660. doi: 10.1371/journal.pone.0272660 (PMC9374266; doi:10.1371/journal.pone.0272660)
Supplement: Attachment — Submitted filename: Response_reviewer.docx [file pone.0272660.s007.docx]

Dear Dr. Logez,

Thank you for submitting your manuscript to PLOS ONE. After careful consideration, we feel that it has merit but does not fully meet PLOS ONE’s publication criteria as it currently stands. Therefore, we invite you to submit a revised version of the manuscript that addresses the points raised during the review process.

Please submit your revised manuscript by Jul 29 2022 11:59PM. If you will need more time than this to complete your revisions, please reply to this message or contact the journal office at [plosone@plos.org](mailto:plosone@plos.org). When you're ready to submit your revision, log on to <https://www.editorialmanager.com/pone/> and select the 'Submissions Needing Revision' folder to locate your manuscript file.

Please include the following items when submitting your revised manuscript:

- A rebuttal letter that responds to each point raised by the academic editor and reviewer(s). You should upload this letter as a separate file labeled 'Response to Reviewers'.
- A marked-up copy of your manuscript that highlights changes made to the original version. You should upload this as a separate file labeled 'Revised Manuscript with Track Changes'.
- An unmarked version of your revised paper without tracked changes. You should upload this as a separate file labeled 'Manuscript'.

If you would like to make changes to your financial disclosure, please include your updated statement in your cover letter. Guidelines for resubmitting your figure files are available below the reviewer comments at the end of this letter.

If applicable, we recommend that you deposit your laboratory protocols in protocols.io to enhance the reproducibility of your results. Protocols.io assigns your protocol its own identifier (DOI) so that it can be cited independently in the future. For instructions see: <https://journals.plos.org/plosone/s/submission-guidelines#loc-laboratory-protocols>. Additionally, PLOS ONE offers an option for publishing peer-reviewed Lab Protocol articles, which describe protocols hosted on protocols.io. Read more information on sharing protocols at <https://plos.org/protocols?utm_medium=editorial-email&utm_source=authorletters&utm_campaign=protocols>.

We look forward to receiving your revised manuscript.

Kind regards,

Hideyuki Doi
Academic Editor
PLOS ONE

Journal Requirements:

When submitting your revision, we need you to address these additional requirements.

1. Please ensure that your manuscript meets PLOS ONE's style requirements, including those for file naming. The PLOS ONE style templates can be found at <https://journals.plos.org/plosone/s/file?id=wjVg/PLOSOne_formatting_sample_main_body.pdf> and 
<https://journals.plos.org/plosone/s/file?id=ba62/PLOSOne_formatting_sample_title_authors_affiliations.pdf>

We have modified the manuscript to meet PLOS ONE’s requirements. We have thus modified the style of the headings, the manner to refer to figures, tables, and to the supplementary material file. Supplementary Material captions and numbering were also changed. Reference numbers were placed in brackets (and not in parentheses).

2. We note that Figure (1) in your submission contain copyrighted images. All PLOS content is published under the Creative Commons Attribution License (CC BY 4.0), which means that the manuscript, images, and Supporting Information files will be freely available online, and any third party is permitted to access, download, copy, distribute, and use these materials in any way, even commercially, with proper attribution. For more information, see our copyright guidelines: <http://journals.plos.org/plosone/s/licenses-and-copyright>.
 
We require you to either (1) present written permission from the copyright holder to publish these figures specifically under the CC BY 4.0 license, or (2) remove the figures from your submission:
 
1. You may seek permission from the original copyright holder of Figure (1) to publish the content specifically under the CC BY 4.0 license. 
 
We recommend that you contact the original copyright holder with the Content Permission Form (<http://journals.plos.org/plosone/s/file?id=7c09/content-permission-form.pdf>) and the following text:
“I request permission for the open-access journal PLOS ONE to publish XXX under the Creative Commons Attribution License (CCAL) CC BY 4.0 (<http://creativecommons.org/licenses/by/4.0/>). Please be aware that this license allows unrestricted use and distribution, even commercially, by third parties. Please reply and provide explicit written permission to publish XXX under a CC BY license and complete the attached form.”
 
Please upload the completed Content Permission Form or other proof of granted permissions as an "Other" file with your submission. 
 
In the figure caption of the copyrighted figure, please include the following text: “Reprinted from [ref] under a CC BY license, with permission from [name of publisher], original copyright [original copyright year].”
 
2. If you are unable to obtain permission from the original copyright holder to publish these figures under the CC BY 4.0 license or if the copyright holder’s requirements are incompatible with the CC BY 4.0 license, please either i) remove the figure or ii) supply a replacement figure that complies with the CC BY 4.0 license. Please check copyright information on all replacement figures and update the figure caption with source information. If applicable, please specify in the figure caption text when a figure is similar but not identical to the original image and is therefore for illustrative purposes only.

We replaced the figure which contained Open Street Map content by a new figure integrating BD ORTHO data from IGN (French National Geographic Institute), that are under a licence ‘Etalab 2.0’. We contacted IGN by email, asking for the right to use the illustration (that was sent attached to the mail) under a licence CC BY 4.0. We were answered that Etalab 2.0 licence is entirely compatible with CC BY 4.0 licence, and that BD ORTHO illustration made with BD ORTHO data can be distributed with a CC BY 4.0 licence as long as IGN and years are mentioned on the picture. Therefore, we modified the figure and added ‘IGN’ mentions and the ‘year’ on each panel. A copy of the conversation (in French) was added at the end of this document. We can also provide the pdf of this conversation.

3. PLOS journals require authors to make all data underlying the findings described intheir manuscript fully available without restriction unless the data are subject to ethical restrictions or owned by someone other than the authors (<https://journals.plos.org/plosone/s/data-availability#loc-acceptable-data-access-restrictions>). Therefore, we ask that you please upload underlying data to an appropriate data repository and update your Data Availability Statement accordingly or provide all contact details for where an interested researcher would need to apply to gain access to the relevant data. Please note that it is not acceptable for an author to be the sole named individual responsible for ensuring data access.

Following PLOS ONE requirements, all the data underlying the statistical analyses were made available in a figshare repository. They will be available if the manuscript would be considered for publication (doi: [10.6084/m9.figshare.20173217](https://doi.org/10.6084/m9.figshare.20173217)) in PLOS ONE and a link toward the manuscript will be done.

4. In your Methods section, please provide additional information regarding the permits you obtained for the work. Please ensure you have included the full name of the authority that approved the field site access and, if no permits were required, a brief statement explaining why.

The following sentences were added at the end of the first section of Material and Methods: ‘Field site access was approved by the water managers of each lake: Communauté de Communes du Lac d'Aiguebelette (CCLA) for lake Aiguebelette, Syndicat Mixte d'Aménagement et de Développement de Serre-Ponçon (SMADESEP) and by Conseil Départemental des Bouches-du-Rhône for lake Etang des Aulnes. No specific authorization were necessary to filter water.’

5. In your Methods section, please include a comment about the state of the animals following this research. Were they released, euthanized or housed for use in further research? If any animals were sacrificed by the authors, please include the method of euthanasia and describe any efforts that were undertaken to reduce animal suffering

The paragraph concerning the gillnets data could have been confusing. Indeed, these fish data are collected by French water authorities, such the Water Agencies and the French Agency for the Biodiversity as part of the monitoring undergo for the Water Framework Directive (WFD). As authors we only have accessed to these data collected in national database, we did not sampled fish nor manipulated them. Our sampling design only concerned the filtration of water to collect eDNA.

This paragraph was modified to make it clearer: ‘We used data obtained from national fishing surveys conducted by the French Biodiversity Agency (OFB) and water agencies as part of the WFD monitoring programme. Fish data were collected according to CEN standard [51].’

6. We note that you have indicated that data from this study are available upon request. PLOS only allows data to be available upon request if there are legal or ethical restrictions on sharing data publicly. For more information on unacceptable data access restrictions, please see <http://journals.plos.org/plosone/s/data-availability#loc-unacceptable-data-access-restrictions>.

In your revised cover letter, please address the following prompts:

a) If there are ethical or legal restrictions on sharing a de-identified data set, please explain them in detail (e.g., data contain potentially sensitive information, data are owned by a third-party organization, etc.) and who has imposed them (e.g., an ethics committee). Please also provide contact information for a data access committee, ethics committee, or other institutional body to which data requests may be sent.

b) If there are no restrictions, please upload the minimal anonymized data set necessary to replicate your study findings as either Supporting Information files or to a stable, public repository and provide us with the relevant URLs, DOIs, or accession numbers. For a list of acceptable repositories, please see <http://journals.plos.org/plosone/s/data-availability#loc-recommended-repositories>.

We will update your Data Availability statement on your behalf to reflect the information you provide

As already mentioned, data supporting all the statistical analyses are now uploaded on figshare and have a DOI ([10.6084/m9.figshare.20173217](https://doi.org/10.6084/m9.figshare.20173217)). They will be completely available without any request, if this manuscript is published. Moreover, in the metadata we will make a link pointing toward the paper URL.

Additional Editor Comments (if provided):

I got the recommendations and comments from an expert reviewer in the field. The reviewer agreed that the manuscript is technically sound and the data support the conclusions.
However, the lack of an explanation in the Methods and Results sections was suggested, and I share the comments. Therefore, I can invite you to submit a revised version of the manuscript that addresses the points raised by the reviewers.


[Note: HTML markup is below. Please do not edit.]

Reviewers' comments:

Reviewer's Responses to Questions

**Comments to the Author**

1. Is the manuscript technically sound, and do the data support the conclusions?

The manuscript must describe a technically sound piece of scientific research with data that supports the conclusions. Experiments must have been conducted rigorously, with appropriate controls, replication, and sample sizes. The conclusions must be drawn appropriately based on the data presented.

Reviewer #1: Yes

2. Has the statistical analysis been performed appropriately and rigorously?

Reviewer #1: Yes

3. Have the authors made all data underlying the findings in their manuscript fully available?

The [PLOS Data policy](http://www.plosone.org/static/policies.action#sharing) requires authors to make all data underlying the findings described in their manuscript fully available without restriction, with rare exception (please refer to the Data Availability Statement in the manuscript PDF file). The data should be provided as part of the manuscript or its supporting information, or deposited to a public repository. For example, in addition to summary statistics, the data points behind means, medians and variance measures should be available. If there are restrictions on publicly sharing data—e.g. participant privacy or use of data from a third party—those must be specified.

Reviewer #1: Yes

4. Is the manuscript presented in an intelligible fashion and written in standard English?

PLOS ONE does not copyedit accepted manuscripts, so the language in submitted articles must be clear, correct, and unambiguous. Any typographical or grammatical errors should be corrected at revision, so please note any specific errors here.

Reviewer #1: Yes

5. Review Comments to the Author

Please use the space provided to explain your answers to the questions above. You may also include additional comments for the author, including concerns about dual publication, research ethics, or publication ethics. (Please upload your review as an attachment if it exceeds 20,000 characters)

Reviewer #1: Comments:
In this study, the authors used eDNA and assessed the spatio-temporal variability of its signal for fish monitoring in lakes.
I suggest publishing the paper after some revisions.

L27: May authors change to "6 weeks" instead of "2 months" (according to Line 154)
On line 27, ‘2 months’ was changed by ‘6 weeks’: ‘oligotrophic lake) every 6 weeks for 1 year’

L32: maybe authors present here the traditional method used for the comparison.
This precision was added in parenthesis on lines 32-33: ‘traditional fish monitoring methods (standardized gillnet samplings)’

L77: May authors change fish to eukaryotic species
This was done on line 77: bacteria to eukaryotic species

L84: change to "The number of species that can be detected"
This was done on line 84: ‘The number of species that can be detected with eDNA’

L95: "focused" instead of "focussed"
‘focussed’ was changed by ‘focused’on lines 95-96: ‘Some studies of lacustrine fish focused on these questions’

L97: "both of the two aspects" instead of "the two apsects together"
This was done on line 97: ‘they consider both of the two aspects.’

L101-102: change to "considering possible changes over a year"
This was done one lines 101-102: ‘of the spatio-temporal variability of eDNA for several types of lakes and considering possible changes over a year’

L127: The subfigures may be named as (a) for Aiguebelette, (b) for Serre-Ponçon, and (c) Etang des Aulnes, instead of 1, 2, 3 which is a bit confusing
The subfigure names and the figure caption have been modified accordingly.

L235-241: This paragraph is results and not material and methods
We agree that this paragraph could have been confusing. As it was written, this paragraph looked like a result of our study, while this was an already known issue of the teleo primers, that was identified during the construction of the reference database (Valentini et al. 2016). The fact that some species could not be differentiated at the species level but at the genus levels was therefore part of the material and method and not of the result section. To avoid this confusion the paragraph was reworded. The last sentence of this paragraph concerning the marine species was moved to the result section (lines 309-311).
lines 242-246: ‘By using teleo primers, the following species are not differentiated [29] and are referred to as the genera: Salvelinus sp., Cottus sp. and Gobio sp. This is also the case for the allochthone acclimated species of Carassius genus. The teleo barcode does not discriminate some species belonging to different genera. They are grouped into ‘complexes’ [26] (see Supplementary Materials).’

L276: The thermal profile of Aiguebelette lake is missing from the Supplementary material.
The thermal profile of Aiguebelette lake was added to the Supplementary material

L279, 285, 340: Define the specific Supplementary Material (eg 1, 2..) in each case
‘Supplementary Material’ was replaced by the specific citation everywhere in the text, line 156 (S1 Table), lines 244-245 (see S2 Text), line 283 (S3 Fig), line 289 (S1 Table), line 348 (but see S4 Fig for the reads), line 404 (all figures in S5 Figs).

L293-295: This paragraph is a bit confusing. Someone cannot understand immediately why authors refer to Carassius carassius and Abramis brama. The first one is an allocthonous species and the reference in the second species is explained in the next sentece, however someone should know that the common bream is Abramis brama. Please be more clear in this paragraph.
We agree that this sentence was not clear, we therefore reworded it (lines 299-300): ‘Surprisingly, although 49 individuals of *A. brama* were captured in 2011, none was found in 2015, excluding common bream from the taxonomic inventory.’

Line 344-345: A comment about what the study of each site reveals (i.e. Fig 4), is missing
The goal pursues by the analysis on all the samples realised, was first to see if there was a distinction of these samples according to the lakes. As the lake was clearly the first factor differentiating the samples (as revealed by the non-overlapping of samples from two different lakes), we considered that the the spatio-temporal dynamics of the eDNA signal should be analysed lake by lake. This is what we tried to express in this paragraph: ‘The NMDS based on all eDNA samples (Bray–Curtis distance, stress = 0.156) showed that the fish fauna of the three sites were significantly different whatever the sampling location and period (PERMANOVA, p < 0.001; Fig. 4) as revealed by the non-overlapping point clouds. Due to these differences, the spatio-temporal dynamics of the eDNA signal was studied for each site independently.’ We think that the results of Fig 4 is detailed enough here.

2 more general comments:
1. In eDNA studies it is common to use technical repetitions for the samples. In this study, the authors filtered a big volume of water and further they evaluated all the results from all the sampling campaigns in each lake. Therefore, I understand why they chose this specific approach. However, authors may explain a bit more why they followed this sampling strategy.
The replication level may be reached through multiple ways (multiple samples per localities, multiple extractions per sample and multiple PCRs per extraction), as demonstrated by Ficetola [1]. We decided that instead of making 30 replicates of 1L per sampling location to use an integrative sampling strategy of 30L in a single filter cartridge, which limits the possibility to sample again exactly at the same place and possibility of 'contamination' between the field replicates. This volume is similar to the total volume of water collected with the single point sampling from other authors to collect the majority of the fish biodiversity [2–4]. In line 161 we explained why we preferred the integrative sampling. ‘This integrative sampling allowed to sample a large variety of habitats and to reduce the species detection biases due to local variation’. We privileged to increase the replication level in the laboratory steps, indeed we performed 12 PCR replicates to be sequenced, as suggested by [1] for rare species detection, and we increased the sequencing depth as it was demonstrated that the number of detected species increase with the sampling depth [5].

1. Ficetola GF, Pansu J, Bonin A, Coissac E, Giguet-Covex C, Barba M, et al. Replication levels, false presences and the estimation of the presence/absence from eDNA metabarcoding data. 2015;15. doi:10.1111/1755-0998.12338

2. Handley LL, Read DS, Winfield IJ, Kimbell H, Johnson H, Li J, et al. Temporal and spatial variation in distribution of fish environmental DNA in England’s largest lake. Environ Dna. 2019;1: 26–39. doi:10.1002/edn3.5

3. Macher T-H, Schütz R, Arle J, Beermann AJ, Koschorreck J, Leese F, et al. Beyond fish eDNA metabarcoding: Field replicates disproportionately improve the detection of stream associated vertebrate species. Metabarcoding Metagenomics. 2021;5: e66557. doi:10.3897/mbmg.5.66557

4. Bylemans J, Gleeson DM, Hardy CM, Furlan E. Toward an ecoregion scale evaluation of eDNA metabarcoding primers: A case study for the freshwater fish biodiversity of the Murray–Darling Basin (Australia). Ecol Evol. 2018. doi:10.1002/ece3.4387

5. Bylemans J, Gleeson DM, Lintermans M, Hardy CM, Beitzel M, Gilligan DM, et al. Monitoring riverine fish communities through eDNA metabarcoding: determining optimal sampling strategies along an altitudinal and biodiversity gradient. Metabarcoding Metagenomics. 2018;2: 1–12. doi:10.3897/mbmg.2.30457

2. It is proved in many eDNA studies that the temperature affects the eDNA. In this study it seems that it is not the case. However, I think that a discussion about this issue is missing.
In this study, we did not explicitly focused on this parameter or test it, but we integrate it in many aspects of the study and of the discussion. First, we provided vertical profile of temperature, to know if lakes were stratified or not, which was shown by Littlefair et al. to play a role on eDNA distribution in lakes. Moreover, when conducting a study over a year, temperature is expected to play an important role on lake functioning (stratification dynamics), on organism’s activities, phenology, and thus on eDNA production and decay. Here, we pointed out the link between fish reproduction periods and eDNA signal; however the design of the study does not allow to fully integrate temperature as an explanatory factor for our eDNA data. Adding a specific discussion on the effects of temperature on eDNA should be a little bit far from the topic of the manuscript, which is not dealing with the relative importance of the different factors affecting eDNA signal. Even if temperature have played a role, it would be difficult to specifically address all the possible direct and indirect effects of temperature on eDNA signal, the data available here do not offer the possibility to quantify these effects. Therefore, even if we recognize the interest of such topic, we cannot add a specific paragraph since we have not the relevant data to address this question; we are more focused on the general question of when and where to sample, in the scope of using eDNA in routine in monitoring programs.

6. PLOS authors have the option to publish the peer review history of their article ([what does this mean?](https://journals.plos.org/plosone/s/editorial-and-peer-review-process#loc-peer-review-history)). If published, this will include your full peer review and any attached files.


If you choose “no”, your identity will remain anonymous but your review may still be made public.


**Do you want your identity to be public for this peer review?** For information about this choice, including consent withdrawal, please see our [Privacy Policy](https://www.plos.org/privacy-policy).

Reviewer #1: No

[NOTE: If reviewer comments were submitted as an attachment file, they will be attached to this email and accessible via the submission site. Please log into your account, locate the manuscript record, and check for the action link "View Attachments". If this link does not appear, there are no attachment files.]

While revising your submission, please upload your figure files to the Preflight Analysis and Conversion Engine (PACE) digital diagnostic tool, <https://pacev2.apexcovantage.com/>. PACE helps ensure that figures meet PLOS requirements. To use PACE, you must first register as a user. Registration is free. Then, login and navigate to the UPLOAD tab, where you will find detailed instructions on how to use the tool. If you encounter any issues or have any questions when using PACE, please email PLOS at [figures@plos.org](mailto:figures@plos.org). Please note that Supporting Information files do not need this step.

*In compliance with data protection regulations, you may request that we remove your personal registration details at any time.* [*(Remove my information/details)*](https://www.editorialmanager.com/pone/login.asp?a=r)*. Please contact the publication office if you have any questions.*

**De:**                                     contact.geoservices <contact.geoservices@ign.fr>

**Envoyé:**                                                vendredi 24 juin 2022 09:47

**À:**                                                            Maxime LOGEZ

**Objet:**                                                            RE : Faire état d’une autre difficulté CRM:0107874

Bonjour,

Il n'y a aucune contre-indications dans les termes de la licence [Etalab 2.0](https://www.etalab.gouv.fr/licence-ouverte-open-licence/) , tant que vous indiquez la paternité et la date de dernière mise à jour de les ressources utilisées comme cela est fait sur votre document.

Cordialement,

       <https://www.ign.fr/espace-presse/ign-changer-dechelle>

Relation Utilisateurs -  Géoservices IGN
Courriel : [contact.geoservices@ign.fr](mailto:contact.geoservices@ign.fr)

Afin de mieux répondre à vos attentes, nous vous invitons à [partager votre avis](https://www.sphinxonline.com/surveyserver/s/etudesmk/Site_Geoservices/questionnaire.htm) sur notre nouveau site Géoservices

Les modalités d'accès aux services web évoluent au 1er février 2022
Cliquez [ici](https://geoservices.ign.fr/actualites/2021-10-04-evolution-des-modalites-dacces-aux-services-web) pour en savoir plus

[Inscrivez-vous](https://geoservices.ign.fr/) à la lettre Géoservices (accessible en bas de page du site)

------------------- Message d'origine -------------------
**De :** pgie.geoservices.p <[pgie.geoservices.p@agriculture.gouv.fr](mailto:pgie.geoservices.p@agriculture.gouv.fr)>;
**Reçu :** Thu Jun 23 2022 17:47:10 GMT+0200 (heure d’été d’Europe centrale)
**À :** contact.geoservices <[contact.geoservices@ign.fr](mailto:contact.geoservices@ign.fr)>;
**Sujet :** Faire état d’une autre difficulté

Informations sur le demandeur :

- Nom : Logez

- Prénom : Maxime

- Organisme : INRAE

- Adresse email : [maxime.logez@inrae.fr](mailto:maxime.logez@inrae.fr)

- Numéro de téléphone :

Description de la demande :

Bonjour, Je voulais savoir si la licence etalab 2.0 était compatible avec la licence CC BY 4.0. Je souhaite publier un article dans une revue en open access (Plos One) qui distribue son contenu sous licence cc BY 4.0. J'ai créer une illustration à partir de données IGN (BD Ortho, voir figure ci-jointe), en spécifiant l'origine des données. Est-ce que je peux la diffuser dans ce cadre (CC BY 4.0) ? Bien cordialement, Maxime Logez

Envoyé par Géoservices.ign.fr

[Contactez-nous](https://www.ign.fr/contact)

Institut national de l'information géographique et forestère (IGN)

73 avenue de Paris 94165 SAINT-MANDÉ CEDEX
